# Inactivation of Histone Chaperone HIRA Unmasks a Link Between Normal Embryonic Development of Melanoblasts and Maintenance of Adult Melanocyte Stem Cells

**DOI:** 10.1111/acel.70070

**Published:** 2025-05-14

**Authors:** Farah Jaber‐Hijazi, Rouven Arnold, Karthic Swaminathan, Kathryn Gilroy, Alexander T. Wenzel, Anthony Lagnado, Kristina Kirschner, Neil Robertson, Claire Reid, Neil Fullarton, Jeff Pawlikowski, Taranjit Singh Rai, Ian Baranyk, Christina Huan Shi, Kevin Y. Yip, Karen Blyth, Jill P. Mesirov, Melissa L. Harris, João F. Passos, Laura M. Machesky, Peter D. Adams

**Affiliations:** ^1^ School of Cancer Sciences University of Glasgow Glasgow UK; ^2^ CRUK Scotland Institute Glasgow UK; ^3^ School of Health and Life Sciences University of the West of Scotland Glasgow UK; ^4^ Sanford Burnham Prebys Medical Discovery Institute La Jolla California USA; ^5^ Faculty of Life Sciences, Centre for Skin Sciences University of Bradford Bradford UK; ^6^ Department of Medicine University of California San Diego (UCSD) La Jolla California USA; ^7^ Moores Cancer Center University of California San Diego California USA; ^8^ Department of Physiology and Biomedical Engineering Mayo Clinic Rochester Minnesota USA; ^9^ Department of Hematology Mayo Clinic Rochester Minnesota USA; ^10^ Northern Ireland Centre for Stratified Medicine Ulster University Ulster UK; ^11^ Department of Biology University of Alabama at Birmingham Birmingham Alabama USA; ^12^ Department of Biochemistry University of Cambridge Cambridge UK

## Abstract

Evidence indicates that the integrity of in utero development influences late life healthy or unhealthy aging; however, specific links between them are unclear. Histone chaperone HIRA is thought to play a role in both life stages, and here, we explore this role using the murine pigmentary system by investigating and comparing the effects of its lineage‐specific knockout, either conditionally during embryogenesis or postnatally. Embryonic knockout of *Hira* in tyrosinase+ neural crest‐derived lineages, including melanoblasts, led to reduced melanoblast numbers during embryogenesis, with single‐cell RNA sequencing analysis indicating evidence of lineage‐specificity defects. This was supported in an in vitro model using melb‐a melanoblasts in which *Hira* knockdown affected lineage identity and melanoblast differentiation potential, with ATAC‐seq data indicating a role of HIRA in orchestrating chromatin accessibility. Interestingly, however, newborn *Hira* knockout mice had wild type numbers of differentiated melanocytes, albeit functionally defective, as demonstrated by very mild hypopigmentation of the first hair coat, increased melanocyte telomere‐associated DNA damage foci, and impaired response to proliferative challenge. Moreover, as they aged, mice with embryonic melanoblast *Hira* knockout displayed marked defects in melanocyte stem cell maintenance and premature hair graying. Importantly, this phenotype was not observed after postnatal inducible knockout, indicating an essential role for HIRA at embryonic stages that is transmitted to adulthood, rather than a direct postnatal requirement within the pigmentary system. This genetic model shows that HIRA function during early development lays a foundation for maintaining lineage identity and subsequent maintenance of adult tissue‐specific stem cells during aging.

## Graphical abstract

Expression of HIRA in embryonic melanoblasts is required for proper epigenetic programing and lineage differentiation. Although HIRA deficiency results in a mild phenotype at birth and young adulthood, the melanocytic lineage is sensitive to proliferative challenge, prone to loss of stem cells and dramatically accelerated hair greying in later life. This shows how early development lays a foundation for maintaining lineage identity and subsequent maintenance of adult tissue‐specific stem cells during aging.

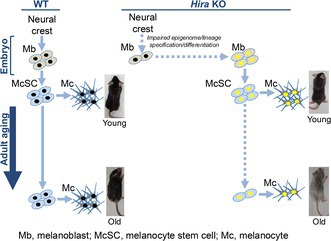

## 1 Introduction

Previous human observational studies have shown that environmental conditions during embryonic development, such as a mother's nutrition or exposure to toxins, can have long‐term effects on health and aging of the offspring (Gluckman et al. 2008). Studies in model organisms also suggest that normal variations in embryonic development can influence adult longevity (Rea et al. 2005; Shindyapina et al. 2022). Epigenetic mechanisms play crucial roles in both development and aging (Sen et al. 2016; Christophersen and Helin 2010; Bell et al. 2019), and have been suggested to be responsible for linking embryonic development to healthy aging and longevity (Tobi et al. 2014).

HIRA is a histone chaperone, specifically targeted to the histone variant H3.3 which differs from H3.1 and H3.2 by only a few amino acids (Tagami et al. 2004). HIRA, which has significant homology to yeast Hir1p and Hir2p proteins, is one of several transcription units identified in the human 22q11 locus (Halford et al. 1993; Lamour et al. 1995). HIRA is a key member of the HIRA histone chaperone complex, also containing UBN1 and CABIN1, and cooperating with ASF1a (Tagami et al. 2004; Rai et al. 2011; Ricketts et al. 2015). At the molecular level, the HIRA histone chaperone complex deposits histone H3.3 into nucleosomes in a DNA replication‐independent manner, mainly at active promoters, enhancers and genic regions (Tagami et al. 2004; Ahmad and Henikoff 2002; Ray‐Gallet et al. 2002; Morozov et al. 2023; Dunjić et al. 2023). Nucleosomes carrying both H3.3 and H2A.Z histone variants are susceptible to disruption, marking active gene promoters (Jin and Felsenfeld 2007). HIRA and histone H3.3 are implicated in the cell response to DNA damage, both playing a role in DNA repair (Adam et al. 2013). It has also been reported that deposition of H3.3 by HIRA at H3.1/H3.3 boundaries helps define the location of early replication zones independent of transcription, maintaining chromatin integrity during these processes (Gatto et al. 2022). In addition, histone H3.3 has also been proposed to play a role in epigenetic memory of a transcriptionally activated state through serial rounds of cell division (Bano et al. 2017; Ng and Gurdon 2008; Szenker et al. 2011).

HIRA is expressed during murine embryogenesis as early as embryonic stages E6.5‐E7.5 during gastrulation (Roberts et al. 2002; Wilming et al. 1997), and ubiquitous expression was observed at E8.5 with higher levels in the cranial neural folds (Wilming et al. 1997). Several studies indicate an essential role of HIRA and H3.3 in oogenesis and embryonic viability and development in mice (Roberts et al. 2002; Inoue and Zhang 2014; Lin et al. 2013; Nashun et al. 2015; Tang et al. 2015; Smith et al. 2022). H3.3 has a repressive role when trimethylated at lysine 27 at developmentally regulated bivalent domains in embryonic stem cells, suggesting an important role in differentiation (Banaszynski et al. 2013). On the other hand, when coupled with H2A.Z at regulatory regions, H3.3 deposition by HIRA leads to poised chromatin states in mouse embryonic stem cells, facilitating transcriptional activation required during differentiation (Yang et al. 2022). HIRA, together with Ubn2, has also been suggested to regulate cell fate determination by suppressing the expression of retrotransposons through deposition of H3.3 and H3K9 methylation (Zhang et al. 2022). Indeed, HIRA has been shown to be required for normal differentiation of various cell lineages, tissues and organs, such as neural cells, cardiomyocytes skeletal muscle and the hematopoietic lineage (Chen et al. 2020; Dilg et al. 2016; Fang et al. 2018; Li and Jiao 2017; Valenzuela et al. 2016; Guo et al. 2022; Esteves de Lima et al. 2021). In addition to roles in differentiation and development, HIRA and H3.3 have also been implicated in cell and tissue aging. For example, HIRA is required for epigenetic integrity in cellular senescence, a stress‐induced proliferation arrest that contributes to tissue aging (Zhang et al. 2005; Rai et al. 2014). H3.3 also accumulates in many tissues with age (Tvardovskiy et al. 2017), including neuronal and glial cells, orchestrating transcriptional programs and maintaining physiological plasticity (Valenzuela et al. 2016; Esteves de Lima et al. 2021; Maze et al. 2015). In sum, HIRA is a histone H3.3 chaperone involved in epigenetic regulation, lineage differentiation, development and aging.

Accordingly, we set out to investigate the role of HIRA in the pigmentary system of mice, a very tractable model to study development and aging mechanisms and the potential links between them. Phenotypes related to abnormal development and aging of the pigmentary system are easy to observe, because defects often lead to a change in the patterns or levels of pigmentation (Hou and Pavan 2008; Bennett and Lamoreux 2003). During embryogenesis, the precursors to melanocytes and melanocyte stem cells (McSCs) are highly proliferative unpigmented cells called melanoblasts. They begin specification from the neural crest at E9.5 and migrate dorsolaterally through the dermis between the somites and to populate the epidermis by E15.5 (Mort et al. 2015). Afterwards, melanoblasts colonize the developing hair follicles (HFs) where they differentiate into melanocytes and McSCs (Mort et al. 2015; Botchkareva et al. 2003). Schwann cell precursors from the glial lineage have also been proposed as a source of melanoblasts, differentiating around E11.5 and migrating along the ventral pathway (Adameyko et al. 2009). In postnatal stages, HFs are in continuous turnover cycles of growth (anagen), regression (catagen) and rest (telogen) (Plikus and Chuong 2008). In all these phases, the HF retains a permanent stem cell rich region, the bulge, but the anagen HF also has a lower, non‐permanent region, the bulb. Hair color is provided by melanin pigments produced from bulb melanocytes, which predominantly differentiate from CD34‐ non‐pigmented McSCs residing in the secondary hair germ region underneath the bulge of telogen HF (Nishimura 2011; Joshi et al. 2019; Rabbani et al. 2011). McSCs are quiescent in the telogen phase but initiate proliferation and differentiation in response to growth signals during the anagen phase (Plikus and Chuong 2008; Nishimura 2011). Progressive loss of McSCs and therefore reduction in melanocyte numbers is one cause of hair graying associated with aging (Nishimura et al. 2005).

Besides the difference in their location or pigmentation, melanoblasts, McSCs and mature melanocytes can be distinguished from each other by gene expression patterns. In brief, before entering the growing HFs, melanoblasts express the master regulator of the melanocytic lineage microphthalmia‐associated transcription factor (MITF), dopachrome tautomerase (DCT), paired box gene 3 (PAX3), tyrosinase (TYR), premelanosome protein (PMEL17), receptor tyrosine kinase (c‐KIT), and SRY‐box transcription factor (SOX10) (Li and Hou 2018; Baxter and Pavan 2003). After entering the HF, increasing expression of MITF and pigmentation genes, such as *Tyr* and *Pmel17*, lead to differentiation into bulb melanocytes (Botchkareva et al. 2003), while diminishing levels of c‐KIT and pigmentation genes lead to the formation of bulge McSCs (Peters et al. 2002).

To probe the role of HIRA in the mouse pigmentary system, we generated a lineage‐targeted knockout mouse model, *TyrCre::Hira* ^*fl/fl*^, in which McSCs, melanocytes, and melanoblasts (and some other neural crest‐derived lineages) are constitutively deficient for HIRA from early in development, and also an inducible knock out model *TyrCreER::Hira* ^*fl/fl*^ in which HIRA can be inactivated in McSCs and melanocytes young adults. One or both models were also crossed to reporters (*Rosa26‐Lox‐STOP‐Lox* (*LSL*)*‐tdTomato* and *Dct::LacZ*) and oncogenic drivers (*TyrNras*) to probe function of HIRA in this lineage. Using these mouse models coupled to single‐cell RNA‐seq and in vitro studies coupled with bulk RNA‐seq and ATAC‐seq, we find that HIRA is required during early and mid‐embryogenesis for proper development of melanoblasts. While defects caused by HIRA inactivation are normalized during late embryogenesis and are barely detectable in melanocytes and McSCs at birth and in young mice, they manifest later as profound defects in McSC maintenance during adult aging. Importantly, these defects are only observed when HIRA is inactivated during embryogenesis, but not in young adults. These results demonstrate a role for HIRA function during early development that lays a foundation for subsequent maintenance of adult McSCs during aging.

## 2 Results

### 2.1 *Hira* Knockout Causes a Reduction in Melanoblast Numbers During Embryonic Development

**FIGURE 1:**
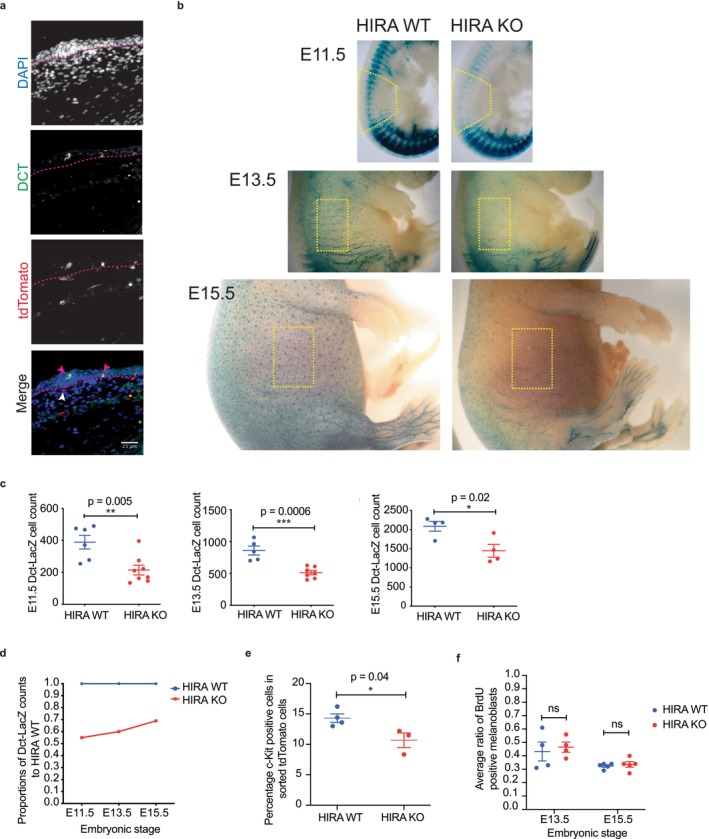
*Hira* knockout causes a reduction in melanoblast numbers during embryonic development. (a) Immunofluorescence in *TyrCre::Hira* ^*wt/wt*^ (HIRA WT) E15.5 mouse embryos showing tdTomato expression, identified by RFP antibody (red), specifically in melanoblasts (magenta arrow heads) in the epidermis identified by DCT stain (green). The epidermis and dermis of the skin are separated with dotted magenta line, epidermis on top. White arrowheads point to non‐melanoblast dermal tdTomato+ cells. Antibody specificity was confirmed by Cre negative or tdTomato negative samples as negative controls. Scale bar: 25 μm. (b) X‐gal stain in E11.5, E13.5, and E15.5 in *TyrCre::Hira* ^*wt/wt*^ (HIRA WT) and *TyrCre::Hira* ^*fl/fl*^ (HIRA KO) *Dct::LacZ* embryos showing melanoblast distribution. Embryos are shown in real relative size. Yellow boxes represent the areas quantified. (c) Quantification of yellow boxes in (b). Error bars represent the standard error of the mean (SEM). Embryo numbers at E11.5, WT *n* = 6, and KO *n* = 8; E13.5, WT *n* = 5, and KO *n* = 7; E15.5, WT *n* = 4, and KO *n* = 4 from at least two litters for each timepoint. (d) Quantification of Dct‐LacZ counts (c) in HIRA KO and HIRA WT normalized to WT at each time point, showing a diminishing difference in the number of HIRA KO to HIRA WT melanoblasts as gestation advances. (e) Percentage of melanoblasts that are c‐KIT positive, selected using a BV711‐conjugated antibody to c‐KIT, within the tdTomato populations in *tdTomato* HIRA WT and HIRA KO E15.5 embryos. Melanoblasts were quantified by FACS profiling of single trunk skin cell suspensions. *tdTomato* HIRA WT, *n* = 4; *tdTomato* HIRA KO, *n* = 3. See Figure S1 for the gating strategy used for quantification. (f) Percentage of BrdU positive melanoblasts quantified by BrdU and DCT antibody stains in E13.5 and E15.5 embryo cross sections following a 2‐h BrdU pulse, with *n* = 4 for each genotype at E13.5 (*p* = 0.7) and *n* = 5 for each genotype at E15.5 (*p* = 0.6). Scatter dot plot data in c, e, and f were analyzed using an unpaired *t‐*test showing mean ± SEM. ns: Non‐significant (*p* > 0.05); *p < 0.05; **p < 0.01 and ***p < 0.001.

**FIGURE 2:**
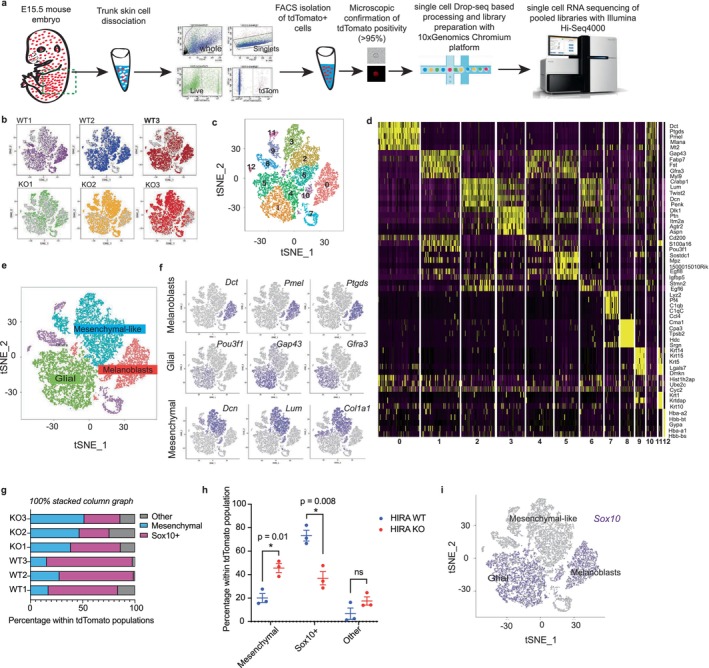
HIRA is required for melanoblast development. (a) Scheme of single‐cell RNA sequencing method. 3 *TyrCre::Hira* ^*wt/wt*^:*tdTomato* (WT) and 3 *TyrCre::Hira* ^*fl/fl*^:*tdTomato* (KO) embryos were individually processed. (b) t‐SNE plots showing clusters based on gene expression among a total of 8655 single cells from 3 WT and 9743 single cells from 3 KO embryos. Cells from all three WT and three KO replicates are superimposed in each plot with the cells of each sample highlighted in a specific color on each of the six plots. (c) t‐SNE plot generated using the Seurat package showing 13 cell clusters (numbered 0–12) based on gene expression relationship from three HIRA WT and three HIRA KO tdTomato+ cells grouped together. (d) Heat map displaying top five distinguishing genes of each of the 13 clusters in (c). (e) t‐SNE plot of all six pooled samples showing three major cell types. (f) t‐SNE plots showing the expression of representative marker genes in the three major cell types. Expression level is color‐coded in purple. (g) 100% stacked column graph displaying the percentage of *Sox10*+ and mesenchymal‐like cells within the tdTomato population in each of the six samples. Cell counts and percentages can be found in Table S5. (h) Scatter dot plots showing the percentages of cells in each of the populations in g in WT versus KO samples, analyzed using an unpaired *t‐*test showing mean ± SEM. *p > 0.05. ns: non‐significant (*p* > 0.05). (i) t‐SNE plot displaying *Sox10* expression level in melanoblasts and glial cells.

*Hira* ^*fl/fl*^ mice (Rai et al. 2014) were crossed with *TyrCreA* (X‐linked) or *TyrCreB* (autosomal) mice in which Cre recombinase is under the control of the *Tyr* gene promoter, allowing inactivation of *Hira* in the melanocytic lineage as early as embryonic days E9.5–10.5 (Delmas et al. 2003). The generated *TyrCre::Hira* ^*fl/fl*^ mice were in turn crossed with *Rosa26‐Lox‐STOP‐Lox* (*LSL*)*‐tdTomato* mice to track Cre recombinase activity (Madisen et al. 2010). Recombination efficiency was confirmed in E15.5 embryos by co‐localization of DCT, a melanoblast marker, and tdTomato in the epidermis (Figure 1a). While all observed Dct + cells were tdTomato+ in all examined samples (*n* > 3), not all tdTomato+ cells are DCT+ because, as reported previously (Delmas et al. 2003), the *Tyr* promoter also directs expression in some other non‐melanocytic lineages (Figure 1a; also see Figure 2). These results confirm *TyrCre*‐directed recombination in embryonal DCT+ cells.

To investigate a role for HIRA in DCT+ embryonic melanoblasts, we crossed *TyrCre::Hira* ^*fl/fl*^ mice with *Dct::LacZ* reporter mice (Mackenzie et al. 1997) in order to track melanoblasts through embryonic development. We compared melanoblast number and distribution between *TyrCre::Hira* ^*fl/fl*^ *Dct::LacZ* (HIRA KO) and control *TyrCre::Hira* ^*wt/wt*^ *Dct::LacZ* (HIRA WT) at E11.5, E13.5 and E15.5 of embryogenesis, by staining with X‐gal. This showed that HIRA KO embryos have a significant reduction in the number of melanoblasts compared to HIRA WT embryos at all three stages (Figure 1b,c). Interestingly, the ratio of melanoblast numbers in HIRA KO to that in HIRA WT was 0.55 at E11.5, 0.59 at E13.5 and 0.69 at E15.5, suggesting a greater effect of HIRA KO at earlier stages of embryogenesis (Figure 1c,d). We also quantified melanoblast numbers by flow cytometry (Figure S1a). To identify melanoblasts, we stained cell suspensions from individual *LSL‐tdTomato* HIRA KO and WT E15.5 embryo skins (Figure 1a) with a BV711‐conjugated antibody against c‐KIT, widely used as a melanoblast cell‐surface marker (Harris et al. 2018; Yonetani et al. 2008). We found that the percentage of c‐KIT+ tdTomato+ cells was significantly reduced in HIRA KO samples (10.7% ± 2%, *n* = 3) compared to HIRA WT samples (14.3% ± 1.4%, *n* = 4) (Figure 1e), indicating a reduction in melanoblast numbers, consistent with that observed in the *Dct‐LacZ* reporter system.

To determine whether HIRA had an effect on melanoblast proliferation that might account for their decreased number in HIRA KO embryos, E13.5 and E15.5 embryos were harvested after a 2‐h BrdU pulse to pregnant dams. Cross sections were stained with anti‐BrdU and anti‐DCT antibodies to determine the percentage of proliferating melanoblasts in both the upper and lower halves of the body along the axial plane. The average percentage of BrdU positive melanoblasts was not significantly different between HIRA KO and HIRA WT embryos; (46.5% ±7.7% (*n* = 4) in HIRA KO and 43.4% ± 14.2% (*n* = 4) in HIRA WT embryos at E13.5; 33.7% ±4.7% (*n* = 5) in HIRA KO and 32.5% ±2.1% (*n* = 5) in HIRA WT embryos at E15.5) (Figure 1f and Figure S1b). In sum, at E13.5–15.5, embryonic HIRA KO melanoblasts show similar proliferation to WT melanoblasts, but reduced total number.

### 2.2 HIRA Is Required for Melanoblast Development

To better understand the defect in melanoblast development in HIRA KO embryos, we isolated tdTomato+ cells from trunk skin of *TyrCre::Hira* ^*wt/wt*^:*tdTomato* (WT) and *TyrCre::Hira* ^*fl/fl*^:*tdTomato* (KO) E15.5 embryos (three embryo replicates each genotype). The purified cells were 95% tdTomato+ (Figure 2a and Figure S2a). Transcriptomes of WT and KO melanoblasts were compared by single‐cell RNA sequencing (scRNAseq) on the 10× Chromium platform (see Table S1 for details on the samples). Data were analyzed by 10× Cell Ranger, cLoupe and Seurat (Satija et al. 2015). Comparing data from the individual WT and KO replicates to the t‐SNE plot generated from all pooled WT and KO samples showed that the cell subclusters in WT and KO samples were broadly similar (Figure 2b).

Based on the expression of subcluster‐specific marker genes and biological or functional pathways such as the cell cycle, 13 subclusters (numbered 0–12) were observed in the pooled WT and KO cells (Figure 2c,d). However, when only characteristic lineage‐specific genes were considered, we identified three main populations of cells resembling melanoblasts, glial cells and mesenchymal‐like cells with chondroblast, fibroblast and osteoblast characteristics (Figure 2e,f). These three major subclusters were distinguished by distinct gene expression profiles: For example, melanoblasts expressed *Dct*, *Pmel*, and *Ptgds*; glial cells *Pou3f1*, *Gap43*, *Gfra3*; and mesenchymal‐like cells *Dcn*, *Lum*, *Col1a1* (Figure 2f). More examples of cluster‐specific distinguishing genes were generated from WT populations only and can be found in Tables S2–S4 and Figure S2b–d. In WT embryos, a minority of tdTomato+ cells were mesenchymal‐like (Figure 2g,h and Table S5), consistent with previous reports that some mesenchymal cells are derived from the neural crest (Simoes‐Costa and Bronner 2015). Since scRNAseq shows that these cells do not express *Tyr* at E15.5 (Figure S2b), presumably these cells are derived from a neural crest lineage that previously expressed *Tyr* to irreversibly activate expression of *tdTomato*. More specifically, Schwann cell precursors of the glial lineage, also previously expressing *Tyr*, may differentiate into mesenchymal cells as well as melanoblasts (Adameyko et al. 2009; Xie et al. 2019). In WT embryos, the majority of tdTomato+ cells were *Sox10*+ melanoblasts and glial cells (74.7%; Figure 2g–i and Table S5). This is consistent with a neural crest‐derived SOX10+ progenitor as a precursor to both melanoblasts and glial cells (Mort et al. 2015), and the previously demonstrated activity of the *Tyr* promoter in these cell types (Delmas et al. 2003; Haninec and Vachtenheim 1988; Tief et al. 1996).

Although tdTomato+ cells from WT and KO embryos displayed broadly similar cell subclusters, we observed a change in proportions from WT to KO (Figure 2g–i, Figure S2e,f and Table S5). Mean melanoblast percentage was significantly reduced from 30.3% ± 6.2% in HIRA WT to 12.5% ± 4.6% in HIRA KO, in line with our previous observations by flow cytometry analysis and the *Dct‐LacZ* reporter (Figure 1b–e). When we assessed combined *Sox10*+ cells (melanoblasts and glial cells), we observed a significant reduction in the mean *Sox10+* population from 73.22% ± 8% in HIRA WT to 36.9% ± 9.8% in HIRA KO samples (Figure 2g,h). These data are also in agreement with the downregulation in expression of various other melanoblast and glial‐specific genes, such as *Dct*, *Ptgds, Fabp7* and *Gfra3*, *Mpz* and *Mbp* as observed in differential expression (DE) analysis of melanoblast and glial populations separately or combined (Tables S6–S9). The decrease in *Sox10*+ cells in KO populations was accompanied by a significant increase in the percentage of mesenchymal‐like cells (20.1% ± 6.6% in HIRA WT to 45.6% ± 6.6% in HIRA KO) (Figure 2g,h, Figure S2e,f and Table S5). This was accompanied by an upregulation of the mesenchymal genes *Col1a1*, *Col3a1*, *Lum*, and *Dcn* as observed from DE analysis of KO against WT melanoblast clusters (Table S6). Moreover, the KO mesenchymal‐like population had an increased expression of epithelial‐to‐mesenchymal transition (EMT) genes (Figure S2g), indicating that WT and KO cells were qualitatively distinct. Therefore, loss of HIRA not only decreases the proportion of tdTomato+ cells with a melanoblast identity, but the *Sox10*+ lineage as a whole, accompanied by a corresponding increase in atypical mesenchymal‐like cells.

**FIGURE 3:**
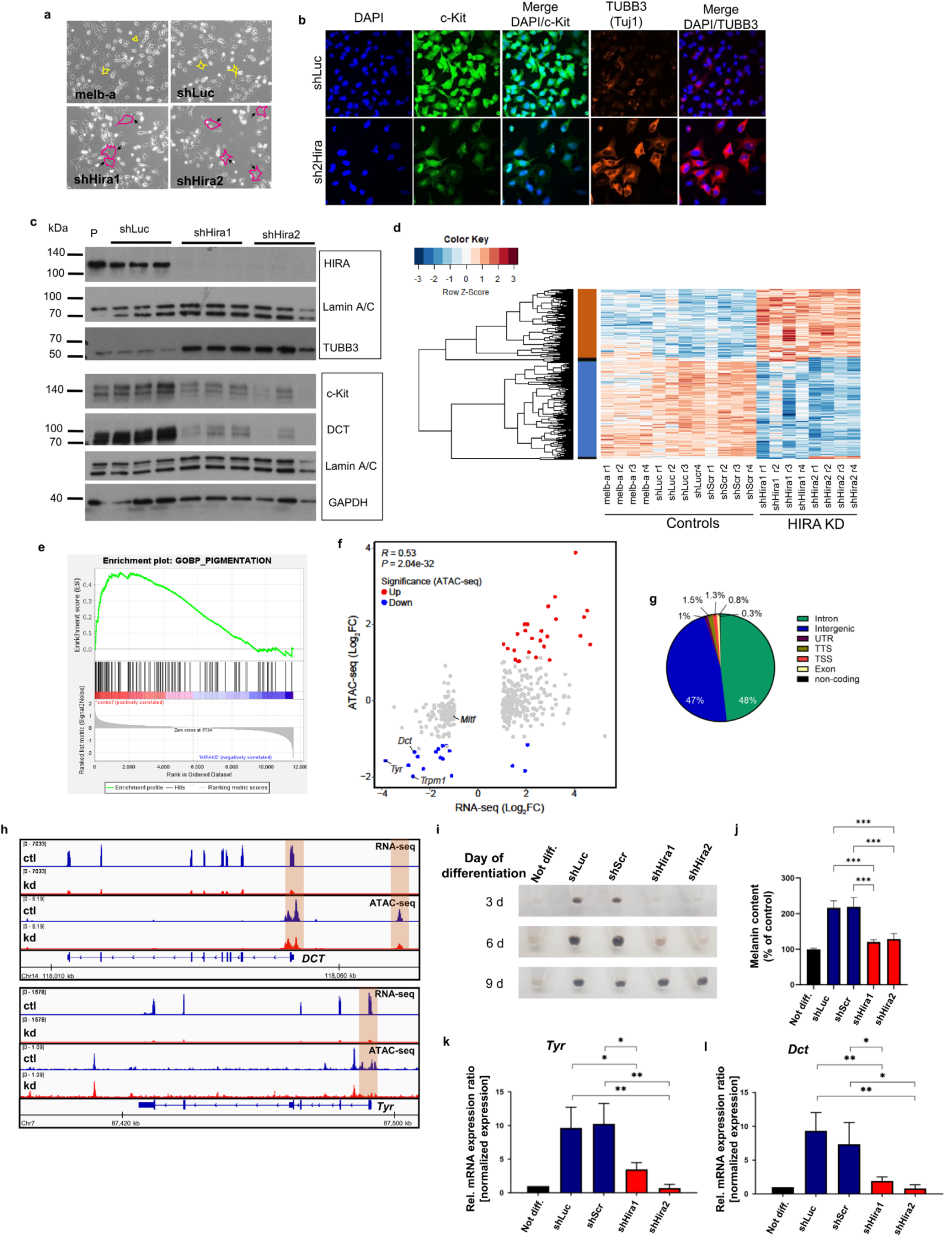
HIRA maintains lineage identity of melb‐a cells and is essential for their differentiation. (a) Brightfield images of representative melb‐a cells 1 week following infection with shHira1 and shHira2 lentiviral vectors, with shLuc as control. Three independent replicates each (three culture plates each infected separately). Uninfected (non‐drug‐selected) parental cells are labeled as melb‐a. Black arrows indicate enlarged cells (also traced in red) compared to parental and shLuc control (some cells traced in blue for comparison). Magnification 10×. (b) c‐KIT (green) and TUBB3 (red) immunofluorescence on melb‐a cells 10 days post‐infection. Scale bars: 25 μm. (c) Representative Western blots of HIRA, TUBB3, and the melanoblast‐specific proteins c‐KIT and DCT in lysates from shLuc, shHira1, and shHira2 melb‐a cells, three independent replicates each, 10 days following infection and drug selection. GAPDH and Lamin A/C were used as loading and sample integrity controls, respectively. Blots performed in the same gel or a re‐probed membrane are boxed together. Molecular weights were marked using Spectra multicolor broad range protein ladder. P: parental. (d) Heat map of RNA‐seq analysis of control (prental melb‐a, shLuc, and shScrambled) vs. shHira1 and shHira2 knockdown (KD) melb‐a cells. The cells were harvested 1 week after lentiviral infections, from *n* = 4 independent experiments. The cluster separates the differentially expressed genes by color with positive or negative row‐scaled Z‐scores represented in red and blue, respectively. (e) Gene set enrichment analysis (GSEA) revealed a decreased expression of pigmentation genes in HIRA KO melb‐a cells. Vertical bars refer to individual genes in the pigmentation gene set, and their position reflects the contribution of each gene to the enrichment score. (f) Dispersion plot showing the correlation between differential gene expression and differential ATAC peaks. (g) Genomic distribution of differently accessible ATAC peaks between both controls and shHIRA samples. (h) RNA‐seq and ATAC‐seq profiles in the genomic loci of DCT (upper panel) and Tyr (lower panel). (i) Timeline of pelleted melb‐a melanoblasts after 3, 6, and 9 days of differentiation, induced by NDP‐MSH, into melanocytes. Non‐differentiated cells were cultured in normal melb‐a growth medium and are indicated as not diff. (j) Melanin quantification of melb‐a cells after 4 days of differentiation as measured spectrophotometrically at 405 nm. (k, l) Quantitative real‐time PCR analysis of tyrosinase (k) and DCT (l) after 3 days of differentiation. Relative expression was normalized to the expression of GAPDH. Graphs show mean ± SD (*n* = 3, *p < 0.05, **p < 0.01, ***p < 0.001).

To confirm these results in a complementary model in vitro, we examined the consequence of *Hira* knockdown in mouse melb‐a melanoblasts (Sviderskaya et al. 1995). For this, we used two different lentivirus‐encoded shRNAs to knockdown *Hira*, shHira1, and shHira2 (Rai et al. 2014), and analysis was performed 7–10 days following infection and drug selection. Uninfected (parental) cells and cells expressing an shRNA targeted to luciferase (shLuc) were used as negative controls. Initial phenotypic observations indicated that when compared to both control lines, shHira1 and shHira2 cells appeared to be larger, with larger nuclei, (Figure 3a and Figure S3a). Knock down of expression of HIRA protein was confirmed by western blot, and this was accompanied by downregulation of another member of the HIRA chaperone complex, CABIN1, as previously reported (Rai et al. 2011) (Figure S3b). When we assessed melanoblast/cyte markers, we found a striking reduction in the expression of c‐Kit, Dct and MITF, and also the melanoblast/glial marker Sox10, in line with the scRNAseq data (Figure 3b,c and Figure S3c,d). On the other hand, we found an increase in the expression of the TUBB3 protein (TUJ1) (Figure 3b,c), but no effect on other tubulin isoforms (Figure S3e). Expression of TUBB3 has been previously associated with neural, melanocytic and EMT fates (Li and Jiao 2017; Locher et al. 2013; Menezes and Luskin 1994; Sobierajska et al. 2016). Increased expression of the *Tubb3* mRNA was also observed in scRNAseq DE analysis both in melanoblast and glial KO vs WT clusters (Tables S6–S9), demonstrating the specificity of these observations. When we examined a selection of H3 histone modifications, no changes were observed (Figure S3f,g). Also, consistent with data from mouse embryos (Figure 1f), knockdown of *Hira* had no significant effect on DNA synthesis as analyzed by expression of proliferating cell nuclear antigen (PCNA) and EdU pulse labelling, with no signs of cell death (Figure S3e,h,i).

To better understand the apparent loss of melanoblast identity caused by *Hira* knockdown, we performed bulk RNA sequencing (RNA‐seq) of melb‐a cell lines, shHira1 and shHira2, compared to controls, shLuc, shScrambled, and parental. Principal component analysis shows a clear separation of *Hira* knockdown samples from all controls (Figure 3j). Altogether, we identified 281 genes showing an upregulation in the *Hira* knockdown samples, whereas 379 genes were downregulated (Figure 3d, Table S10). Gene ontology analysis of upregulated genes showed enrichment of pathways associated with morphogenesis and migration, while GO analysis of downregulated genes included pathways related to pigmentation and myelination (Figure S3k). The observation of a dysfunctional pigmentation pathway was further confirmed by gene set enrichment analysis (GSEA) (Figure 3e). Using Ingenuity Pathway analysis (IPA) we identified the top upstream transcription regulators of dysregulated genes, namely *Mitf*, *Tp53*, *Tcf7l2*, *Ppargc1a*, *Smarca2*, *Srebf1*, and *Pdlim11* (Figure S3l). Genes expressing the master regulator of melanoblasts, MITF, along with TCF7L2, regulating the Wnt signaling pathway, were found to be suppressed upstream transcription factors. This suggests that both major pathways integral to melanocyte differentiation/melanogenesis were dysregulated. Mechanistically, these expression changes are likely caused by alterations in chromatin regulation. To identify how HIRA impacts chromatin structure, we performed an assay for transposase‐accessible chromatin sequencing (ATAC‐seq). In general, we observed a significant positive correlation between differential gene expression and differential ATAC‐seq signal (Figure 3f). This suggests a causative relationship between chromatin remodeling and the resulting gene expression. The chromatin accessibility of *Hira* knockdown samples indicated a higher number of downregulated peaks (8291) compared to upregulated peaks (5970) (Figure S3m). The genomic distribution of all differently accessible peaks showed that 95% are within introns and intergenic regions, and 1.3% are at TSS (Figure 3g). This suggests that HIRA regulates the chromatin structure of melanoblasts more widely than exclusively at TSS, for example at distal regulatory elements/enhancers consistent with previous reports (Tagami et al. 2004; Ahmad and Henikoff 2002; Ray‐Gallet et al. 2002; Morozov et al. 2023; Dunjić et al. 2023). Examining the genes encoding two of the main melanocytic markers, *Dct* and *Tyr*, we further demonstrated that decreased expression is associated with closed chromatin along the promoters and potential enhancers (Figure 3h). This observation was confirmed by another GO analysis of genes featuring decreased chromatin accessibility at TSS compared to the control samples, which once again identified pathways associated with pigmentation and melanogenesis (Figure S3n).

The RNA‐seq and ATAC‐sec data indicate that the loss of HIRA leads to the impairment of the identity of melb‐a cells as precursors to pigment cells. We therefore asked directly whether the ability of these cells to differentiate into pigmented melanocytes was impaired. To do this, differentiation was induced by NDP‐MSH hormone as described previously (Sviderskaya et al. 2001). Melb‐a cells growing in differentiation medium showed a time‐dependent increase in melanin accumulation (Figure 3i). However, we observed significantly reduced melanin levels in *HIRA* knockdown cells compared to those in the control cells after 4 days of differentiation (Figure 3j), although the levels were observed to be recovered by the 9th day (Figure 3i and Figure S3o). *Tyrosinase* and *DCT* expression levels of *Hira* knockdown samples were reduced concomitantly with the observed lack of melanin production (Figure 3k,l). These results indicate that differentiation or pigmentation is substantially impaired in melb‐a cells as a result of *Hira* knockdown. In conclusion, consistent with the in vivo data, these in vitro data show that HIRA is required for the proper development of the melanoblast lineage, and HIRA inactivation leads to an aberrant differentiation program.

### 2.3 Embryonic Knockout of *Hira* Does Not Affect the Distribution and Number of Melanocytes and McSC Function in Newborn Mice

**FIGURE 4:**
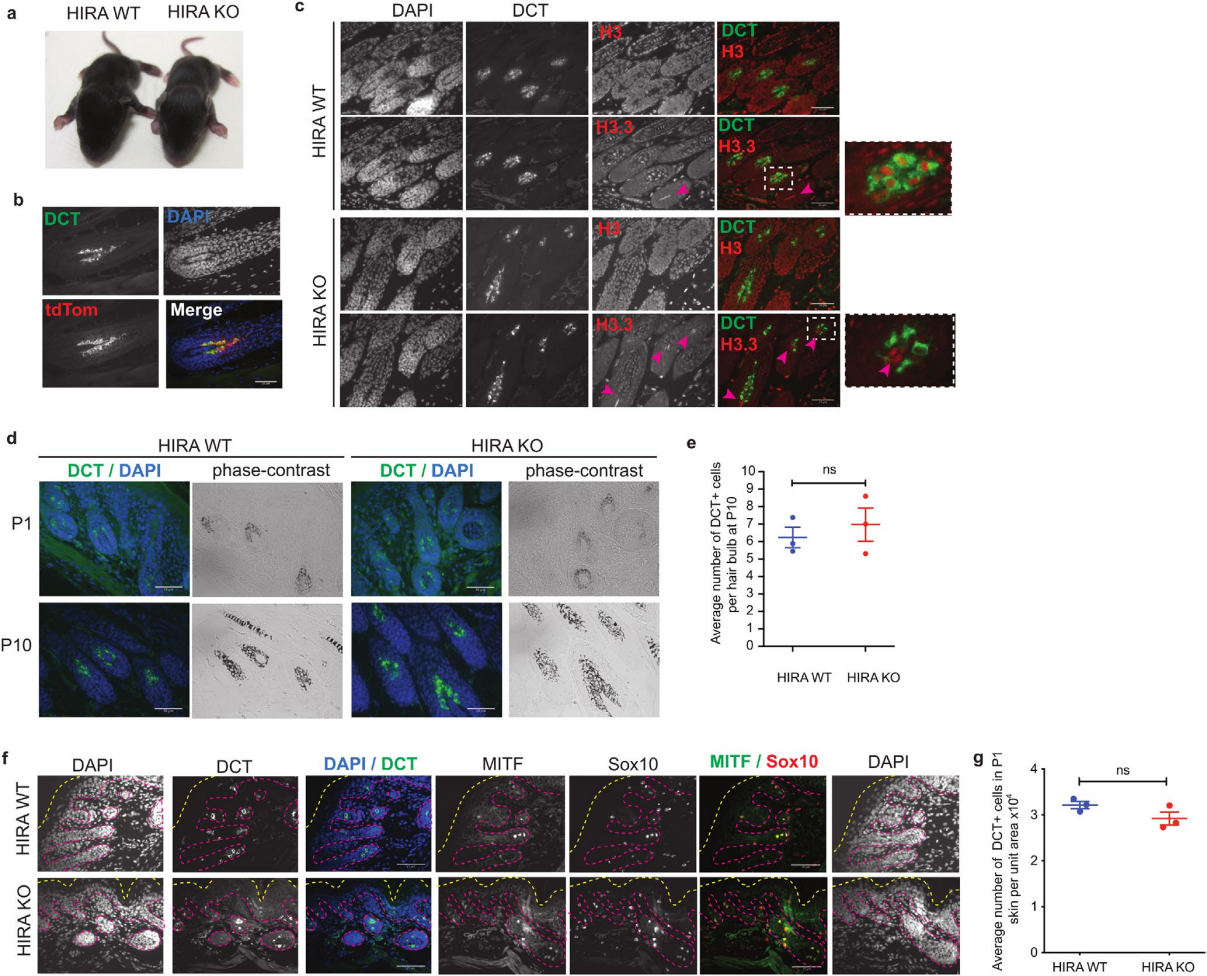
Knockout of *Hira* does not affect the distribution and number of melanocytes and McSC function in newborn and young adult mice. (a) Images of *TyrCre::Hira* ^*wt/wt*^ (HIRA WT) and *TyrCre::Hira* ^*fl/fl*^ (HIRA KO) P11 pups showing coat color of first hairs. (b) Efficient recombination in HIRA KO mice as shown by the overlapping expression of tdTomato (red), induced by Cre‐ recombinase, with DCT+ hair bulb melanocytes (green). Skin sections display hair at the anagen phase of pups at postnatal day 10 (P10). Scale bar: 20 μm. (c) Distinct expression of H3.3 (red) in specific cell types, including melanocytes (green) and dermal papillae (magenta arrow heads), in HIRA WT skin, as opposed to Histone H3 (red), which is more homogeneous in all skin cells. H3.3 staining in melanocytes is lost upon HIRA KO, but not in dermal papillae, while histone H3 expression remains unaffected. Skin longitudinal sections from P10 pups. Scale bar: 20 μm. (d) DCT (green)/DAPI (blue) immunohistochemistry and corresponding phase contrast images showing pigmented melanocytes in growing hair bulbs of HIRA WT and HIRA KO mouse skin at P1 and P10. Scale bars 20 μm. (e) Scatter dot plot showing the average number of melanocytes, measured by DCT positivity, in the hair bulbs from P10 HIRA WT and HIRA pup skin. *n* = 3 pups per genotype; *p* = 0.7. An average of 11–12 hair bulbs were counted pair sample. (f) Representative image of melanocytic distribution in HIRA WT and HIRA KO P1 skin using three melanocytic markers: DCT, MITF, and SOX10. DCT/DAPI and MITF/SOX10/DAPI images are taken from immunofluorescence done on consecutive paraffin sections. The outer layer of the epidermis is traced by a dotted yellow line. Basement membrane and hair follicles are traced by a dotted magenta line. Scale bar: 25 μm. (g) Quantification of DCT positive cells, displayed in (f), per unit area (pixel^2^) of a skin field of view at 20× magnification. *n* = 3 samples per genotype; *p* = 0.1. Scatter dot plot data in e and g were analyzed using an unpaired *t‐*test showing mean with ± SEM. ns: non‐significant (*p* > 0.05).

In light of this clear impediment—but not block—to proper development of HIRA‐deficient melanoblasts, we next set out to understand the consequences for McSC and melanocytes in newborn mice and young adults. Surprisingly, given markedly impaired development of the melanoblast lineage during early embryogenesis, *TyrCre::Hira* ^*fl/fl*^ (HIRA KO) and *TyrCre::Hira* ^*wt/wt*^ (HIRA WT) pups showed only very slight loss of color of their first hairs, both in *TyrCreX* and *TyrCreB* models (Figure 4a), indicating the presence of near‐normally functioning melanocytes at this stage. To confirm *TyrCre*‐directed recombination efficiency in newborn mice, we used the tdTomato reporter system and found that DCT+ melanocytes in anagen HFs of postnatal day 10 (P10) pups uniformly and specifically expressed tdTomato (Figure 4b). In addition, as a functional readout for HIRA inactivation, we used immunofluorescence to assess the nuclear levels of H3.3. Interestingly, in HIRA WT mice, we observed marked enrichment of H3.3 specifically in DCT+ melanocytes and anatomical dermal papillae of hair bulbs, compared to other cells (Figure 4c). Both of these cell types are non‐proliferative (Driskell et al. 2011; Van Neste and Tobin 2004), consistent with DNA replication‐independent deposition of H3.3. However, enrichment in melanocytes was absent in HIRA KO melanocytes (Figure 4c and Figure S4a), but retained in dermal papillae, consistent with lineage‐specific HIRA inactivation in melanocytes and confirming a requirement for HIRA for deposition of H3.3 in these cells. Consistent with near‐normal hair coat color of HIRA KO mice (Figure 4a), immunostaining of melanocytic cells using anti‐DCT antibody confirmed a similar distribution and number of DCT+ cells between HIRA WT and HIRA KO in P1 and P10 pups (Figure 4d). Quantitation confirmed no significant difference in the mean number of melanocytes per anagen HF in P10 HIRA WT (6.24 ± 1 DCT+ cells, *n* = 3 pups) and HIRA KO (6.97 ± 1.6 DCT+ cells, *n* = 3 pups) (Figure 4e). We made similar observations in P1 pup skin using MITF and SOX10 immunostaining in consecutive sections to DCT stains (Figure 4f). Quantification of DCT+ cells in P1 pup skin, a mixture of melanoblasts in the epidermis and McSCs and differentiating melanocytes in newly forming HFs, also showed that their numbers per unit area (x10^−4^) were not significantly different between HIRA WT (3.2 ± 0.14, *n* = 3) and HIRA KO (2.9 ± 0.25, *n* = 3) skins (Figure 4g). Our observations in newborn mice were consistent following the first real anagen at 4 weeks of age (Figure S4b,c) where HIRA KO mice maintained their coat color and DCT+ cell distribution, with only a mild hypopigmentation, when compared to HIRA WT mice. Quantitation of melanin levels in the anagen hair bulbs of 10‐week‐old HIRA WT and KO mice, revealed a slight but significant decline in mean and minimum gray values for the HIRA KO mice, demonstrating a subtle yet measurable reduction in pigmentation (Figure S4d–f). In conclusion, we did not detect a marked effect of embryonic knockout of *Hira* in the melanocytic system on melanocyte number and distribution in newborn mice and young adults, aside from a very slight coat hypopigmentation.

### 2.4 *Hira* Knockout Melanocytic Cells Fail to Respond to Proliferative Challenge

**FIGURE 5:**
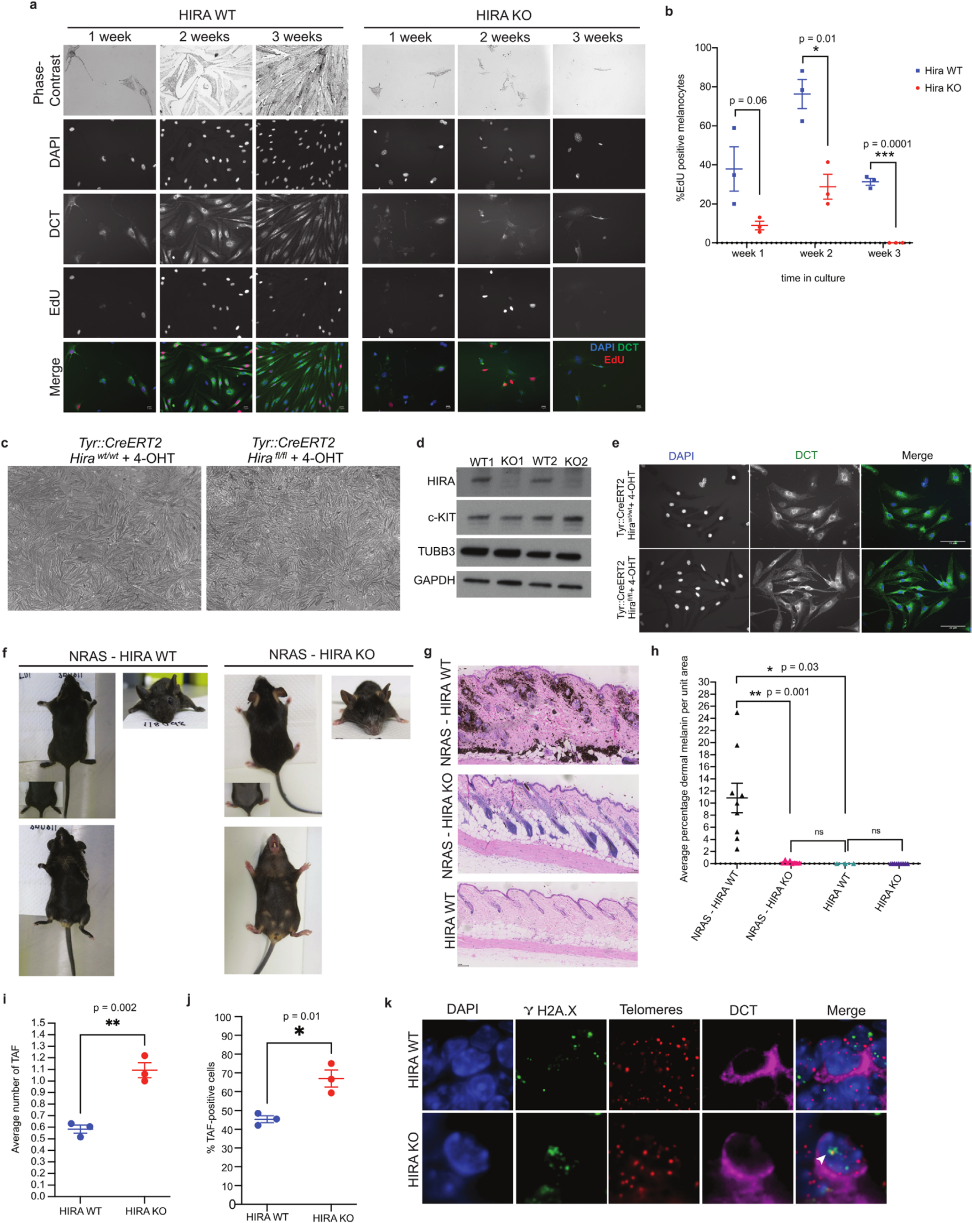
Increased TAF in HIRA knockout melanocytes and failure to thrive under stressful conditions. (a) Comparison of ex vivo growth of primary melanocytes cultured from back skin of *TyrCre::Hira* ^*wt/wt*^ (HIRA WT) and *TyrCre::Hira* ^*fl/fl*^ (HIRA KO) P3 pups at 1, 2, and 3 weeks following seeding. Melanocytes were identified both by pigment and DCT positivity (green). EdU positive nuclei (red) were used to quantify proliferation. Scale bar: 5 μm. (b) Scatter dot plots showing the percentage of EdU positive nuclei. Proliferation was assessed by measuring the percentage of EdU positive nuclei in melanocytes after a 48‐h EdU pulse. Cells were fixed at 1 week, 2 weeks, or 3 weeks post culture. Cells were not passaged during the study period. *n* = 3 independent biological replicates per genotype cultured from different pups. (c) Ex vivo melanocyte cultures from *TyrCreERT2* system with *Hira* ^*wt/wt*^ or *Hira* ^*fl/fl*^, obtained from P1 pups and induced with 4‐hydroxytamoxifen (4‐OHT) at the same time of seeding. Images were taken 3 weeks following culture and prior to any passage. (d) Western blot for c‐KIT and TUBB3 proteins in cells from (c) and biological replicates (WT1 and KO1 were littermates and WT2 and KO2 were littermates from a separate litter). (e) DCT immunofluorescence (green) of induced ex vivo *TyrCreERT2::Hira* ^*wt/wt*^ *and TyrCreERT2::Hira* ^*fl/fl*^ cells fixed after 2 weeks in culture. Scale bar: 25 μm. (f) Images of 5‐month‐old *TyrNras::TyrCre::Hira* ^*wt/wt*^ (NRAS‐HIRA WT) and *TyrNras::TyrCre::Hira* ^*fl/fl*^ (NRAS‐HIRA KO) mice displaying differences in the color of hair and skin of the snout, ears, chin, back, belly, feet, and tail. (g) H&E of longitudinal skin sections displaying difference in pigmentation among NRAS‐HIRA WT, NRAS‐HIRA KO, and HIRA WT skins. (h) Scatter dot plots showing the percentage of dermal melanin per unit area of skin. Quantification of skin melanization in the dermis was measured by calculating the percentage area covered in brown pigment using an ImageJ plugin designed for this purpose. Only the dermal region was quantified; hair follicles, fatty regions epidermis were excluded. Number of mice used for each genotype: NRAS‐HIRA WT, *n* = 9; NRAS‐HIRA KO, *n* = 8; HIRA WT, *n* = 3; HIRA KO, *n* = 10. (i) Average number of telomere‐associated DNA damage foci (TAF) in DCT positive cells (21–35 cells per sample) in P1 pup skins from three HIRA WT and three HIRA KO samples. k, Percentage of TAF positive melanocytes per genotype. j, Representative images of H2A.X immuno‐FISH (Green, H2A.X; red, telomeres) in the nuclei of melanocytes (magenta). White arrowhead: Co‐localization of H2A.X and telomeres indicating TAF. Scatter dot plot data in b, h, i, and j were analyzed using an unpaired *t‐*test showing mean ± SEM. ns: non‐significant (*p* > 0.05; *p < 0.05; **p < 0.01 and ***p < 0.001).

So far our results showed that newborn and young adult HIRA KO mice have seemingly normal functioning McSCs and similar numbers of melanocytic cells as WT mice, despite having reduced melanoblast numbers in earlier developmental stages. To better compare the phenotype and function of newborn WT and HIRA KO melanocytes, we isolated these cells from P1‐P3 pups and cultured them ex vivo, as previously described in a medium containing the mitogen tetradecanoylphorbol‐12‐acetate (TPA) (Woodham et al. 2017). While HIRA WT melanocytes, identified by pigment and DCT expression, showed steady growth over 3 weeks in culture, HIRA KO melanocytes, although attached to the culture plate, consistently failed to expand in number (Figure 5a). Moreover, after a 48 h pulse of EdU following 1, 2, or 3 weeks in culture, fewer HIRA KO melanocytes incorporated EdU compared to HIRA WT, consistent with their inability to expand in number (Figure 5a,b). This indicates a melanocyte functional defect in HIRA KO not previously observed in newborn and young adult mice in vivo, and only revealed when the cells are challenged to proliferate under ex vivo conditions after TPA administration.

Interestingly, this proliferative defect was not observed when HIRA was inactivated postnatally. Conditional inactivation of *Hira* in vitro by the addition of 4‐hydroxytamoxifen (4‐OHT) to melanocytes isolated from *TyrCreERT2::Hira* ^*fl/fl*^ newborn mice had no observable effect on cellular growth (Figure 5c), in contrast to constitutive knockout melanocytes (Figure 5a). Moreover, the expression of Dct, c‐KIT and TUBB3 was not affected in induced *TyrCreERT2::Hira* ^*fl/fl*^ cells when compared to *TyrCreERT2::Hira* ^*wt/wt*^ cells under the same conditions (Figure 5d,e). Recombination and the loss of HIRA in *TyrCreERT2::Hira* ^*fl/fl*^ cells was confirmed by PCR and Western Blot (Figure S5a,d, respectively). This shows that the defect observed in postnatal constitutive HIRA KO *TyrCre::Hira* ^*fl/fl*^ melanocytes necessarily originates in embryonic melanoblasts.

In order to challenge proliferative capacity of the embryonic KO melanocytic cells in vivo, we used the *TyrNras* mouse model in which *Tyr*‐driven expression of an *Nras* ^*Q61K*^ oncogene leads to hyperpigmentation of the skin due to excessive postnatal proliferation of bulb melanocytes and their expansion into dermal regions of the skin (Ackermann et al. 2005). While the hyperpigmentation phenotype was observed as expected in *TyrNras::TyrCre::Hira* ^*wt/wt*^ (NRAS‐HIRAWT) mice, it was clearly suppressed in *TyrNras::TyrCre::Hira* ^*fl/fl*^ (NRAS‐HIRA KO) mice, as observed particularly in the snout, chin, ears, tail, feet and shaved skin (Figure 5f). Hematoxylin and eosin (H&E) stained longitudinal skin sections also showed that the dermal pigmentation observed in NRAS‐HIRA WT was not observed in NRAS‐HIRA KO mice (Figure 5f), and indeed in the latter dermal pigmentation was substantially and significantly reduced to levels similar to HIRA WT mice (Figure 5f–h and Figure S5b). The difference in distribution of DCT+ cells in NRAS‐HIRA KO cell compared to NRAS‐HIRA WT skin was be observed in pups as young as P10/11 where the loss of *Hira* suppressed the expansion of DCT+ cells into the dermis of the skin (Figure S5c). Our findings confirm that decreased pigmentation in NRAS‐HIRA KO compared to that in NRAS‐HIRA WT mice reflects the failure of HIRA KO cells to respond to NRAS‐induced proliferative challenge.

We reasoned that this impaired response of newborn HIRA KO melanocytes to proliferative challenge both in vitro and in vivo might reflect elevated molecular stress in these cells. Specifically, telomeric dysfunction is known to limit the proliferative capacity of cells (Lee et al. 1998; Wright and Shay 1992). Therefore, we examined a marker of telomere dysfunction, telomere‐associated DNA damage foci (TAF) (Victorelli et al. 2019), by measuring the number of H2A.X foci at telomeres in DCT+ cells of P1 pups. We found that the mean number of TAFs per DCT+ cell was 0.58 ± 0.06 in HIRA WT but was significantly higher in HIRA KO cells (1.09 ± 0.11) (Figure 5i). Moreover, the percentage of TAF+ DCT+ cells was also significantly higher in HIRA KO pups (67.01% ± 7.82%) than HIRA WT pups (45.35% ± 3.2%) (Figure 5j,k), indicating a correlation between impaired proliferative capacity and TAFs. Together, these data indicate that, despite their normal numbers and regenerative capacity in the first anagen, newborn HIRA‐deficient McSCs and melanocytes exhibit features of molecular stress and impaired proliferation potential when challenged both in vitro and in vivo.

### 2.5 HIRA Is Specifically Required During Embryonic Development for Melanocyte Stem Cell Maintenance in Adults and Suppression of Hair Graying During Aging

**FIGURE 6:**
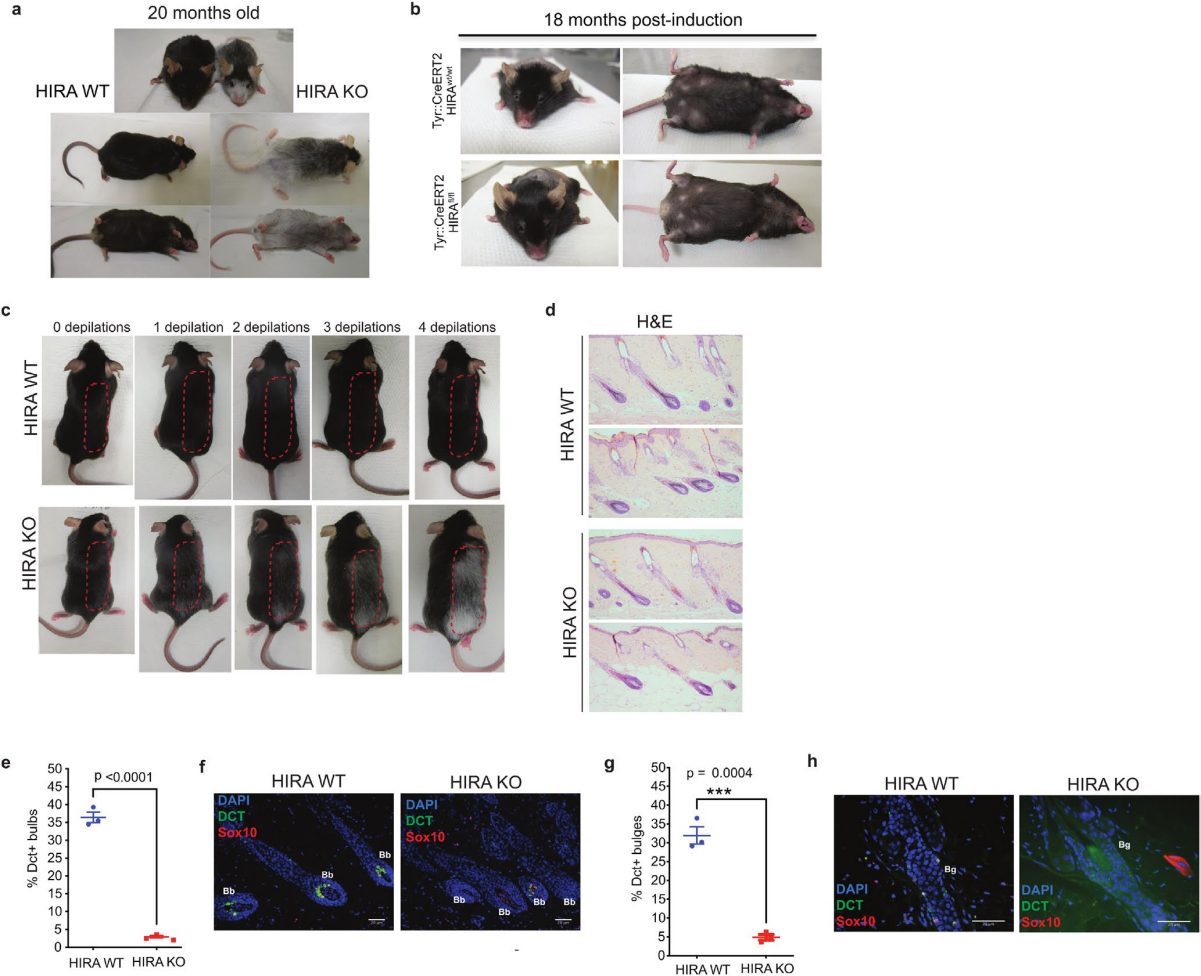
HIRA is required during embryonic development for McSC maintenance in adults and suppression of hair graying during aging. (a) Back, belly, and frontal images (top to bottom) of 20‐month‐old mice showing premature hair graying in *TyrCre::Hira* ^*fl/fl*^ (HIRA KO) mice as compared to *TyrCre::Hira* ^*wt/wt*^ (HIRA WT) mice which retained their dark coat color. (b) Representative images of *TyrCreERT2::Hira* ^*wt/wt*^ *and TyrCreERT2::Hira* ^*fl/fl*^ mice 18 months following tamoxifen induction at 6 weeks of age. Three mice per genotype were observed. (c) Progressive hair graying and loss of pigment in the depilated side of the back skin in HIRA KO mice, while HIRA WT mice retained hair pigment. Images were taken before the first depilation and after full hair growth following each round of depilation, usually every 3 weeks. Four rounds of depilation were performed in total over a 3‐month period, starting with 8‐week‐old mice; representative of *n* = 8 mice. (d) H&E stain of longitudinal skin sections from 13‐month‐old HIRA WT and HIRA KO mice. Skins were harvested 2 weeks following depilation to synchronize hair cycles. (e) Quantification of differentiated melanocytes in the skins of mice in (e) by measuring the percentage of DCT/SOX10 positive hair bulbs. A total of 220–230 anagen hair bulbs were counted per genotype; *n* = 3 mice each. (f) Representative images of hair follicles quantified in (e). Scale bar: 20 μm. (g) Quantification of McSC content in the same mice in (e). A total of 300–400 hair follicles (anagen and telogen phases) were counted for the presence of DCT/SOX10 positive cells in the bulge region. Scatter dot plot data in (e) and (g) were analyzed using an unpaired *t‐*test showing mean ± SEM. ***p < 0.001. (h) Representative images of bulges counted in (g). Scale bar: 20 μm.

Prolonged McSC function and cyclical proliferation is necessary for maintenance of McSCs and regeneration of mature differentiated melanocytes through each hair cycle, and hence suppression of hair graying over the lifecourse (Nishimura et al. 2005). Based on our results, we hypothesized that HIRA‐deficient McSCs would be unable to perform these functions over the long term, despite the apparent normal function of the pigmentary system in newborn mice and young adults (Figure 4). Indeed, consistent with this idea, by 20 months old, naturally aged HIRA KO mice showed a strong premature hair graying phenotype with pigment loss most notable in the snout, belly and lower parts of the back (Figure 6a), with hair graying being gradual (Figure S6a).

Importantly, this premature hair graying phenotype was not observed even 18 months after *Hira* inactivation by 4‐OHT administration to 6‐week‐old conditional KO *TyrCreERT2::Hira* ^*fl/fl*^ mice (Figure 6b). In this case, recombination was confirmed, both in McSCs and melanocytes, by tdTomato expression (Figure S6b,c). This is consistent with the results obtained from ex vivo culture of embryonic KO *TyrCre::Hira* ^*fl/fl*^ and postnatal KO *TyrCreERT2::Hira* ^*fl/fl*^ cells, showing that the ex vivo proliferative defect and the premature hair graying in vivo both depend on embryonic KO of Hira.

To directly probe the function of adult McSCs derived from constitutive/embryonic melanoblast Hira KO, we performed sequential rounds of depilation beginning on HIRA WT and KO mice at 8 weeks of age. Indicative of a McSC functional defect, HIRA KO mice showed progressive but marked hair graying over 4 cycles of depilation (Figure 6c). The effects of depilation were particularly marked in older mice. We aged HIRA WT and KO mice to 13 months and then again challenged the McSCs by depilation. Coat color of these mice exhibited signs of a premature hair graying phenotype after only 1 cycle of depilation (Figure S6d), and H&E longitudinal skin sections showed loss of pigment in HIRA KO anagen HFs compared to HIRA WT (Figure 6d). We quantified differentiated melanocytes and McSCs in these 13 month old mice, identified by DCT and SOX10 positive cells in the hair bulb and bulge, respectively. Strikingly, while in HIRA WT mice 36.4% ± 2.5% of hair bulbs contained melanocytes, only 2.95% ± 0.6% of hair bulbs contained melanocytes in HIRA KO mice (Figure 6e,f). Moreover, the percentage McSC‐containing bulges of telogen and anagen HFs of HIRA KO mice was 4.9% ± 1.3%, significantly lower than that in in HIRA WT mice (32% ± 4%) (Figure 6g,h). We did not find evidence for premature differentiation, a cellular phenotype of McSC aging (Nishimura et al. 2005), in the remaining McSCs in HIRA KO hair bulges, as these cells were neither pigmented nor expressed high levels of MITF, which is generally upregulated with melanocyte differentiation (Figure S6e–g). Our results indicate that HIRA is required during embryonic development for McSC maintenance and suppression of hair graying during aging.

## 3 Discussion

Previous human observational studies have shown that environmental conditions during embryonic development, such as a mother's nutrition or exposure to toxins, can have long‐term effects on the health and aging of the offspring (Gluckman et al. 2008). Studies in model organisms also suggest that normal variations in embryonic development can influence adult longevity (Rea et al. 2005; Shindyapina et al. 2022). Consistent with these previous observations, we showed in this study that a specific developmental abnormality occurring in utero results in barely perceptible defects at birth and young adulthood but marked deficiencies under stress and/or during aging. Specifically, the knockout of the gene encoding histone chaperone HIRA in migratory neural crest cells had a strong impact on melanoblast numbers early in mouse embryonic development, although the melanocytic cell number recovered by birth and the function of melanocyte stem cells appeared normal in young adults. However, these cells failed to respond to growth signals under stress, and later in adult life, McSCs were prematurely depleted, and hair pigment was prematurely lost.

Our data in the melanocytic lineage, both in vitro and in vivo, support the essential role of HIRA for normal cell differentiation and development. Previous studies have shown that HIRA is required for gastrulation, patterning of mesoendodermal cells, and embryonic viability (Roberts et al. 2002; Szenker et al. 2012), and proper differentiation of ES cells (Banaszynski et al. 2013), muscle cells (Esteves de Lima et al. 2021; Yang et al. 2011), hematopoietic cells (Chen et al. 2020), neurogenesis (Li and Jiao 2017), and oogenesis (Nashun et al. 2015). Extending these observations, we show here that HIRA is required for normal embryonic development of SOX10+ melanoblasts and expression of melanocytic genes, *Dct* and *Mitf*. In HIRA KO mice, a decrease in SOX10+ melanoblasts was accompanied by a reciprocal increase in mesenchymal‐like cells, including an abnormal population with features of EMT, perhaps indicative of a switch from a melanoblast to a mesenchymal‐like phenotype. Consistent with this idea, other studies have previously shown a switch of melanocytic to mesenchymal lineages, particularly on downregulation of SOX10 (Lionarons et al. 2019; John et al. 2011), as is observed here. HIRA has been shown to physically interact with PAX3, a transcription factor known to play an essential role in melanoblast specification (Magnaghi et al. 1998). Whether the role of HIRA in the melanocytic lineage is dependent on H3.3 deposition or interaction with other proteins is a crucial question for future studies. At this time, our observations in P10 HFs in which H3.3 abundance is reduced in HIRA KO compared to HIRA WT indicate that HIRA's role in the melanocytic lineage is likely connected to H3.3, and that DAXX, another H3.3 histone chaperone (Choi et al. 2024), does not compensate for HIRA loss. HIRA is known to be involved in H3.3 deposition in euchromatin, such as actively transcribed gene bodies and regulatory elements, while DAXX governs H3.3 deposition in heterochromatin, such as pericentromeres, retrotransposons, and subtelomeric regions (Goldberg et al. 2010). Similarly, our observations in melb‐a cells indicate that HIRA orchestrates chromatin accessibility at TSS and other sites, and therefore, it is likely that the HIRA KO phenotype is caused by H3.3 depletion, similar to its role in skeletal muscle lineage (Esteves de Lima et al. 2021). Subsequently, these chromatin changes lead to transcriptomic alterations that push the melb‐a cells into an altered cellular state in which differentiation stimuli cause delayed pigmentation.

Despite the consistent decrease in the number of melanoblasts in E11.5–E15.5 HIRA KO embryos, newborn and young HIRA KO mice exhibited essentially normal numbers of melanoblasts, McSc, and melanocytes, normal pigmentation, and normally functioning McSC. Consistent with this, the melanoblast deficit in embryos showed a trend to a decrease from E11.5 to E15.5, although this was not significant, at least with these cohort sizes. There are several possible mechanisms by which melanoblasts might recover their numbers during late embryogenesis. First, HIRA KO melanoblasts could compensate for their reduced number during early embryogenesis by increased proliferation in late embryogenesis. However, at E13.5 and E15.5, there was no difference in BrdU incorporation in WT and HIRA KO melanoblasts, suggesting that this is likely not the case, although we cannot exclude a late proliferative burst. Second, HIRA KO melanoblasts might compensate for their reduced number during early embryogenesis by decreased apoptosis in late embryogenesis. Consistent with this possibility, during normal embryogenesis, a proportion of melanoblasts is culled by developmental apoptosis, ~0.5%–1% melanoblasts at E12.5 (Wehrle‐Haller et al. 2001; Silver et al. 2013). Third, HIRA KO mesenchymal‐like cells might undergo a compensatory reversion back to melanoblasts, perhaps because the initial switch to the mesenchymal program is incomplete. Consistent with this third possibility, the HIRA KO mesenchymal‐like population contains a population that is distinct from HIRA WT and expresses genes indicative of EMT, perhaps suggesting only an immature “meta‐stable” mesenchymal‐like phenotype (Nieto et al. 2016). Fourth, the Schwann cell precursors could also contribute to the compensation mechanism by differentiating into melanocytes in late embryogenesis (Adameyko et al. 2009). Future studies will investigate the molecular and cellular mechanisms underlying the recovery of melanoblast numbers during late embryogenesis, perhaps with the use of a reporter system that is more specific to melanoblasts, such as Dct‐GFP (Marie et al. 2020).

Despite superficially normal numbers, phenotype, and function of melanocytic cells in newborn and young adult mice, several lines of evidence show a functional deficit in HIRA KO melanocytic cells. First, in ex vivo assays, HIRA KO cells from newborn mice are unable to proliferate and differentiate into pigmented melanocytes. Second, KO of *Hira* suppresses the hyperpigmentation of *TyrNRasQ61K* mice, which we and others have previously shown is due to a marked postnatal proliferative expansion to generate an abnormal abundance of pigmented melanocytes in the dermis (Ackermann et al. 2005; Pawlikowski et al. 2013). Third, adult HIRA KO mice turn prematurely gray after repeated rounds of depilation or physiological aging, accompanied by depletion of McSCs. Together, these results suggest that HIRA KO melanocytic cells, melanoblasts, and McSCs are impaired in their ability to sustainably proliferate and/or self‐renew when challenged. Importantly, this phenotype depended on embryonic inactivation of *Hira*, suggesting that the defect reflects aberrant programming during early stages of lineage development and/or is accumulated during embryogenesis. There are at least two likely explanations for this. First, DCT+ cells from HIRA KO P1 neonates contained significantly more TAFs than those from WT mice. This may result from increased proliferation, and hence telomere erosion and/or uncapping, during late embryogenesis as the HIRA KO melanoblasts recoup their numbers. Regardless of their origin, TAFs are often associated with cell senescence and aging (Victorelli et al. 2019; Ogrodnik et al. 2017; Anderson et al. 2019; Herbig et al. 2006; Hewitt et al. 2012, 2016; Birch et al. 2015; Fumagalli et al. 2012), including in skin melanocytes (Victorelli et al. 2019). Therefore, the TAFs in neonate and young adult melanocytic cells might limit their proliferative/self‐renewal capacity, ultimately leading to McSC exhaustion and premature hair graying. Second, histone H3.3, and by extension HIRA, is implicated in epigenetic memory through cell division (Bano et al. 2017; Ng and Gurdon 2008; Szenker et al. 2011). Therefore, defects in HIRA‐mediated histone H3.3 deposition during embryo development may translate into “silent” epigenetic defects in newborn mice, but progressive defects in epigenetic memory, gene expression, and cell fate determination through sequential rounds of cell division during adulthood, and hence premature loss of McSCs and hair graying. Consistent with this model, hair bulb melanocytes are particularly enriched in histone H3.3, suggesting a specialized function for histone H3.3 in melanocytes. Because Schwann cell precursors from the SOX10+ glial lineage have been shown to give rise to melanocytes in adult mouse skin (Adameyko et al. 2009), and because we showed that the loss of HIRA in the *Tyr‐cre* system that we used (Delmas et al. 2003) affected the entire SOX10+ population, it is also possible that the progressive premature hair graying in aging mice was exacerbated by this defect in SOX10+ cells (Zhang et al. 2020). Future studies will test these possible mechanisms of adult McSC depletion.

In sum, these studies reveal a role for HIRA in proper differentiation and development of mouse embryo melanoblasts. These results also demonstrate that significant abnormalities in embryonic development can be superficially normalized by birth, only to manifest in later life as defects in the maintenance of adult tissue function. These results highlight the potential connections between proper in utero development of tissue stem and progenitor populations and phenotypes characteristic of late‐life healthy aging.

## 4 Methods

### 4.1 Mice

*Hira* ^*fl/fl*^, *TyrCreA*, *TryCreB*, and *TyrCreERT2* mice were C57Bl/6. *TryCre::Hira* ^*fl/fl*^ mice were generated by crossing *Hira* ^*fl/fl*^ (Rai et al. 2014) with *TyrCre* (Delmas et al. 2003) mice. They were then crossed with either *DCT::LacZ* (Mackenzie et al. 1997), *LSL‐tdTomato* (Madisen et al. 2010), or Tyr‐*N‐RAS* ^*Q61K*^ (Ackermann et al. 2005) reporter mice for melanoblast or melanocyte tracking experiments. For the conditional knockout of *Hira*, embryo experiments were performed with mice carrying the *TryCreB* allele, whereas all postnatal experiments were done with mice carrying the *TryCreA* allele, for which only males were studied. Mice were genotyped both by PCR analysis and Transnetyx (www.transnetyx.com). Test and littermate controls were used in all embryo experiments and many other experiments. *TyrCreERT2* mice (Dhomen et al. 2009; Yajima et al. 2006) were induced by applying 2 mg/mL of Tamoxifen (Sigma) on the shaved back skin of 6‐weekweek‐old mice once a day for 5 consecutive days. Depilation of back skin hair of adult mice was performed using Veet Easy‐Gelwax strips under short anesthesia with isoflurane. All experiments were carried out under the UK Home Office guidelines at the CRUK Scotland Institute biological services and research units.

### 4.2 Fluorescence Immunohistochemistry

Shaved back skin from postnatal mice was fixed with 10% neutral buffered formalin (NBF), embedded in paraffin, and then cut longitudinally along the direction of hair growth. Embryos were harvested after a 2 h BrdU (Sigma GERPN201) pulse to the mother, then fixed in 10% NBF overnight (ON) and transversally cut and embedded back to back in paraffin with heads or tails down. For immunohistochemical (IHC) staining, paraffin sections were dewaxed in xylene (2 x 5 min), then rehydrated in serial ethanol washes (2 x 100%, 95%, and 70%), 2 min each, followed by 5 min in water. Antigen retrieval was performed by boiling the samples in target retrieval solution (Dako S1699), pH = 6, for 25 min, followed by slow cooling at room temperature (RT). After rinsing in water, blocking solution (1% bovine serum albumin (BSA, ThermoFisher Scientific BPE1605‐100) and 5% donkey serum (Sigma D9663) in PBS) was added to tissue sections for 1 h at RT, followed by ON incubation in a closed humid box at 4°C with primary antibodies (Table S11) diluted in blocking solution containing 0.5% Tween‐20 (Sigma P7949). The following day, tissue sections were washed 2  × 5 min with 0.5% Tween‐20/PBS (TPBS), then incubated with secondary antibodies (Table S12) diluted 1/500 in blocking solution with 0.5% Tween‐20 for 40 min in the dark at RT. They were then washed 2 x 5 min with TPBS, stained with 1 μg/mL DAPI (Sigma 9542) and mounted in ProLong gold antifade (ThermoFisher Scientific P36934). Most images were taken using a Nikon Eclipse 80i microscope fitted with a Hamamatsu ORCA‐ER digital camera (C4742‐80) or Nikon A1R confocal microscope.

### 4.3 Immuno‐FISH (TAF Assay)

For FFPE tissues, immunohistochemistry was carried out following an overnight incubation with rabbit monoclonal anti‐γH2AX (1:200, 9718; Cell Signaling); sections were incubated with a goat anti‐rabbit biotinylated secondary antibody (1:200, PK‐6101; Vector Labs) for 30 min at room temperature. Following three PBS washes, tissues were incubated with avidin DCS (1:500, SA‐A‐2011‐1; Vector Labs) for another 30 min at room temperature. Sections were then washed three times in PBS, blocked for 1 h, and incubated overnight with DCT (goat, Santa Cruz sc‐10,451, 1:200); sections were washed three times with PBS, then incubated for 1 h with a donkey anti‐goat 647 (1:1000, Catalog # A‐21447, Invitrogen) followed by three PBS washes and cross‐linked by incubation in 4% paraformaldehyde in PBS for 20 min. Sections were washed in PBS three times and then dehydrated in graded cold ethanol solutions (70%, 90%, 100%) for 3 min each. Tissues were then allowed to air‐dry prior to being denatured in 10 μL of PNA hybridization mix (70% deionized formamide) (Sigma), 25 mM MgCl_2_, 1 M Tris pH 7.2, 5% blocking reagent (Roche) containing 2.5 μg/mL of Cy‐3‐labeled telomere‐specific (CCCTAA) peptide nucleic acid (Panagene), then denatured for 10 min at 80°C and incubated for 2 h at room temperature in a dark‐humidified chamber to allow hybridization to occur. Sections were washed in 70% formamide in 2× SCC for 10 min, followed by a wash in 2× SSC for 10 min, and a PBS wash for 10 min. Tissues were then mounted using ProLong Gold Antifade Mountant with DAPI (Invitrogen). Sections were imaged using in‐depth Z stacking (a minimum of 40 optical slices with 63× objective) followed by Huygens (SVI) deconvolution.

### 4.4 X‐Gal Staining of Embryos

Embryos carrying the *DCT::LacZ* reporter gene were harvested at E11.5, E13.5, or E15.5, fixed in ice‐cold 0.25% glutaraldehyde for 30 min, and stained as previously described (Woodham et al. 2017). In brief, embryos were washed in ice‐cold PBS and then permeabilized in a solution containing 0.02% NP‐40, 2 mM MgCl_2_, and 0.01% sodium deoxycholate in PBS for 30 min at RT. Embryos were then stained with X‐gal solution containing 1 mg/mL X‐gal (Promega V3941), 0.01% sodium deoxycholate, 0.02% NP‐40, 5 mM K_4_Fe(CN)_6_, and 5 mM K_3_Fe(CN)_6_ for 3 nights (E15.5 and E13.5 embryos) or overnight (E11.5 embryos) at 4°C, rotating in the dark. Stained embryos were then washed in PBS, post‐fixed in 10% NBF ON at 4°C, and then stored in 70% ethanol. Images were taken using a Zeiss Stemi 2000‐C dissection microscope fitted with a Canon EOS Rebel 1000D camera, and stained cells were quantified using ImageJ.

### 4.5 tdTomato Positive Cell Sorting and Single‐Cell RNA Sequencing

Embryos carrying tdTomato reporter were harvested at E15.5 and assessed for tdTomato positivity under a fluorescent microscope. Skin pieces from ears and tail were used for genomic DNA extraction and PCR analysis to determine *Hira* ^*fl*^ recombination using two forward primers 5AATGGTGCTTGCT TTTGTGG3′ and 5′TGAAGGTATGGAGGACGCTG3′ and one reverse primer 5′GCATTAC TTAATCCCCAGATGC3′. Embryos were washed in ice‐cold HBSS. Heads, limbs, and tails were cut off and trunk skin was peeled using a scalpel and forceps on the lid of a Petri‐dish on ice. Individual skins were rinsed in HBSS then cut and digested in 300 μL of 0.2 U/mL dispase (ThermoFisher Scientific 17105–041) and 0.5 mg/mL DNaseI (Sigma DN25) in HBSS for 45 min at 37°C. Ice‐cold 5 mM EDTA/PBS was then added to skin suspensions up to a volume of 1 mL to stop the digestion. The mix was passed through 18G followed by 21G needles for full dissociation, then filtered through pre‐wet 50 μm filter membranes (Sysmex 04–0042‐2317) into FACS tubes on ice. Cell suspensions were centrifuged at 300 *g* for 5 min at 4°C and then resuspended in 500 μL sorting buffer (1% FBS, 5 mM EDTA and 25 mM Hepes in PBS). For sorting, at least one tdTomato^+^ *Hira* ^*wt/wt*^ and one *Hira* ^*fl/fl*^ individual skins from the same litter were used. tdTomato^−^ skins were used as negative control for gating. 1 μg/mL of DAPI was used as dead cell marker prior to sorting. tdTomato^+^ live cells were sorted using a BD FACSAria sorter, and 12000–20000 cells were collected per skin piece, corresponding to 1%–2% of the whole cell suspension. Approximately 7000–10000 cells were used for single‐cell RNA isolation, cDNA synthesis, and library preparation according to Chromium protocol (10× Genomics Chromium Single‐Cell 3′ Reagent kits 120237, 120236, and 120267). Libraries per experiment were pooled and sequenced on Illumina Hi‐Seq4000. Table S13 shows details of the three WT and three KO biological replicates from three different embryos per genotype used for scRNAseq analysis. WT1 and KO1 are littermates and were processed simultaneously and pooled in the same sequencing lane. WT2, WT3, and KO2 were littermates, and KO3 was from a separate litter but had a simultaneous plug mating as the other three samples. The last four samples were processed simultaneously and sequenced in the same lane.

### 4.6 scRNAseq Analysis Method

Initial data processing was performed using Cellranger version 2.2.0 (10× genomics). A novel mm10 reference genome was created that included tdTomato and Cre sequences using protocols provided by 10× genomics, then count matrices of scRNAseq reads from each individual sample were created using this custom reference genome and the Cellranger “count” function. All six samples were then combined into one experiment using the Cellranger “aggr” function with the setting –normalize = mapped. Next, a counts matrix was created using the R packages dplyr and Seurat (Satija et al. 2015), and filtered such that genes expressed in at least three cells, and cells with at least 100 expressed genes, were retained. The final matrix contained data for 18,090 genes across 18,453 cells, and was annotated with the sample ID, genotype and sex. To create feature plots, Seurat was used as follows. Firstly, the counts matrix was filtered to exclude cells with more than 40,000 UMIs and more than 10% mitochondrial genes. The data were then log‐normalized, variable genes were identified using a logVMR dispersion function, and the data were scaled. Dimension reduction was performed using the “RunPCA” and “RunTSNE” functions, and feature plots were created using the “FeaturePlot” function. Clusters were mapped to cell types using gene expression profiles.

Differential expression analysis of WT vs KO cells in the total cell population and the indicated subsets was performed using The R packages Scater, Scran and MAST (McCarthy et al. 2017; Finak et al. 2015). Briefly, the annotated, filtered counts matrix was converted to a SingleCellExperiment object and then filtered to remove outliers based on the number of median absolute deviations (nmads) from the median as follows: cells with total counts more than 3 nmads from the median, cells with total features more than 4 nmads from the median, and cells with mitochondrial content more than 4 nmands from the median were dropped. Genes with an average expression < 0.1 across all cells were then dropped. The resulting matrix (5985 cells by 5053 genes) was then normalized using the “computeSumfactors” function, and variable genes were identified using a gamma‐distributed GLM. Expression from the 4485 highly variable genes were then correlated, and hierarchical clustering performed using Ward's clustering criterion. Gene expression in the resulting clusters was used to identify the indicated cell populations, and the dataset was trimmed to contain only subsets of clusters, as appropriate. Differential expression analysis was performed using a zero‐inflated regression model from the package “MAST,” and the most significantly changed genes were noted.

### 4.7 Melanoblast Quantification From tdTomato Populations

tdTomato+ skin cell suspensions from E15.5 embryos were prepared and genotyped as described above. After digestion and the initial filtering step, cells were pelleted then resuspended in 2 mL ice‐cold staining solution (0.5% BSA/PBS) and counted using a hemocytometer. Cells were pelleted and resuspended in 200 μL of staining solution, and 1 μL of Fc block (BD Biosciences 553,142) was added per tube for 10 min on ice. In addition to tubes corresponding to individual skins from WT and KO, controls were prepared corresponding to tdTomato‐ cell suspension (unstained), BV711 FMO (fluorescence minus one), and tdTomato FMO. BV711 conjugated CD117 (c‐kit) antibody (BioLegend 105,835) was added to cell suspensions corresponding to WT, KO, and tdTomato FMO tubes at 1 μg per million cells. An additional control corresponding to isotype control (BD Biosciences 563,045) was also used. Cells were incubated with antibodies in the dark for 30 min on ice. 1 mL of staining solution was added per tube, and the cells were pelleted and resuspended in sorting solution for FACS analysis as described above. BV711 positive cells within the tdTomato population corresponded to melanoblasts. Flow cytometry was performed using BD FACSAria. FlowJo was used for gating and cell quantification (Figure S1).

### 4.8 Plasmids, Transfections, and Viral Infections

LRMIP‐shHira1 and LRMIP‐shHira2 (Rai et al. 2014) were used to knock down *Hira* in melb‐a cells, with pLRMIP‐shluc used as a control. Lentiviruses were produced by transfecting 293 T cells with 20 μg of hairpin plasmids, 8 μg of psPAX2 (Addgene), and 4 μg of pLP/VSVG (Invitrogen) using lipofectamine 2000. Following 6 hours, the transfection medium was replaced with fresh culture medium (DMEM containing 10% FBS and 2 mM L‐glutamine). Virus‐containing supernatants were collected 24 h and 48 h following transfections and were used to infect melb‐a cells supplemented with 8 μg/mL of polybrene (Millipore TR‐1003‐G) ON at 37°C. Successfully infected cells were selected using 1 μg/mL of puromycin (Invivogen ant‐pr) in melb‐a culture medium.

### 4.9 Melb‐a Culture, Differentiation, and Melanin Quantification

Melb‐a line (Sviderskaya et al. 1995) was cultured in RPMI medium (Gibco) containing 10% FBS, 2 mM of L‐glutamine, 25 U/mL of penicillin, 25 μg/mL of streptomycin, 20 ng/mL of stem cell factor (SCF) (Sigma S9915), and 40 pM of fibroblast growth factor 2 (Fgf2) (Sigma SRP4038). Cells were grown at 37°C with 5% CO_2_ and passaged when reaching an 80%–90% confluency. Differentiation was induced 24 h after lentiviral transduction using DMEM supplemented with 10% FBS, 2 mM of L‐glutamine, 25 U/mL of penicillin, 25 μg/mL of streptomycin, 200 nM of TPA, and 1 nM of NDP‐MSH. The infected cells were selected using 1 μg/mL of puromycin. The melanin content after 4 days of differentiation was determined spectrophotometrically. In brief, 5 × 10^5^ cells were pelleted and solubilized in 1 mL of 1 N NaOH/10% DMSO for 2 h at 80°C. Samples were centrifuged at 12,000 *g* for 10 min, and the supernatants were transferred to a new tube. The absorbance of the supernatants was measured at 405 nm using a microplate reader. The melanin content was calculated relative to the non‐differentiated melb‐a control cells.

### 4.10 RNA Sequencing and Analysis

RNA was extracted from cells using TRIzol and Direct‐zol RNA MiniPrep Plus kit (Zymo R2072). The quality of RNA was assessed using Tapestation from Agilent Technologies. RNA‐seq libraries were prepared by the Genomics Core at SBP Medical Discovery Institute, La Jolla, following standard Illumina protocols. Poly‐A RNA sequencing was conducted on the Illumina NextSeq500 platform with ~30 million reads per sample.

The code used for the analysis of RNA‐seq data and for the generation of graphs is publicly available (Berry Alexander et al. 2021). In brief, raw fastq files were aligned to mm10 using the Kallisto pipeline. EnsemblDB was used to annotate data, edgeR to normalize read counts, and Limma to determine differentially expressed genes (DEGs). GO analysis for RNA‐seq was performed using ShinyGO (Ge et al. 2020).

### 4.11 ATAC‐Seq

The assay for transposase‐accessible chromatin sequencing was performed using Active Motif's ATAC‐seq kit. In total, 100 000 fresh cells after selection were tagmented as previously described (Buenrostro et al. 2013). Samples were lysed in ATAC‐seq lysis buffer, tagmented, and processed for library generation and purification. The samples were sequenced by the Genomics Core at SBP Medical Discovery Institute, La Jolla, on the Element Biosciences Aviti platform.

After sequencing reads were trimmed and first aligned against chrM (mouse genome version mm10) to discard mitochondrial reads using Bowtie2 (Langmead and Salzberg 2012). Unaligned reads to chrM were subsequently aligned against mouse genome version mm10 (excluding chrM) using Bowtie2 (“‐‐very‐sensitive ‐‐no‐discordant ‐X 2000 ‐p 12”). Multi‐mapping reads were removed, and alignments were shifted using Deeptools alignmentSieve v3.4.3 (Ramirez et al. 2016). Duplicate reads were flagged and removed using Picard MarkDuplicates v2.22.0. MACS2 v2.2.9.1 (Zhang et al. 2008) was used to call peaks in each replicate. Reproducible peaks for replicates (*n* = 2) of each condition were determined using IDR v2.0.4.2 (Li et al. 2011). Peak sets for the two conditions were merged using bedtools v2.29.2 (Quinlan and Hall 2010) and peaks overlapping ENCODE blacklisted regions were excluded. Consensus merged peaks (70,436 peaks) were annotated using Homer annotatePeaks.pl. (Heinz et al. 2010) and peak tags counted. Sample efficiencies (fraction of reads in peaks, FRIP) for the samples ranged from 0.37 to 0.53. Sample FRIP scores were used to normalize and generate ATAC‐seq signal data (bigwig format) using Deeptools bamCoverage. Differential accessibility of peaks were tested using edgeR v3.38.4 (Robinson et al. 2010). Profile plots of signal over selected genomic regions were generated using computeMatrix and plotProfile utilities in Deeptools.

### 4.12 Gene Expression Analysis

RNA was extracted from (~1 × 10 (Bell et al. 2019)) melb‐a cells using TRIzol and Direct‐zol RNA MiniPrep Plus kit (Zymo R2072). RNA quantity and purity were assessed using a spectrophotometer (NanoDrop One^C^, Thermo Fisher). cDNA was prepared from 1 μg of RNA using RevertAid reverse transcriptase (Thermo Fisher EP0441) according to the manufacturer's protocol. All primers were purchased from IDT. *Dct* forward primer: GGA CCG GCC CCG ACT GTA ATC; *Dct* reverse primer: GGG CAA CGC AAA GGA CTC AT; *Tyr* forward primer: CGG CCC AAA TTG TAC AGA GAA GC; *Tyr* reverse primer: CTG CCA GGA GAA GAA GGA TTG. Quantitative real‐time PCR was performed using the PowerUp SYBR Green Master Mix (Thermo Fisher A25742) on the QuantStudio 6 Flex Real‐Time PCR Systems (Applied Biosystems). Amplification signals were all observed between cycles 15 and 30. The fold change was calculated using the 2^(‐ΔΔCt) method, with GAPDH as an endogenous control.

### 4.13 Primary Melanocyte Isolation and Culture

Primary mouse melanocytes were isolated and cultured as previously described (Woodham et al. 2017). Back skin from 1‐, 2‐, or 3‐day‐old pups was peeled and rinsed in PBS, then cut, and incubated in collagenase I + IV (5 mg/mL, ThermoFisher Scientific 17100017 and 17104019) for 40 min at 37°C. Tail tips of pups were sent to Transnetyx for genotyping. After washing in HBSS (ThermoFisher Scientific 14065‐049), skin pieces were incubated with cell dissociation buffer (ThermoFisher Scientific 13151014) for 10 min at 37°C. Skin pieces were then further dissociated using 18G and 21G needles and then washed in HBSS. Finally, cells were resuspended and cultured in F‐12 (Gibco) growth medium containing 10% fetal bovine serum (FBS), 200 nM12‐tetradecanoylphorbol‐12‐acetate (TPA), and 100 μg/mL of primocin (Invivogen ant‐pm‐1) at 37°C with 5% CO_2_. To select against fibroblasts and keratinocytes, cultures were treated for 3 days a week with 50 μg/mL G418 (Formedium). Pure melanocyte cultures were established 6–8 weeks following initial isolation.

### 4.14 Immunofluorescence, EdU Labeling of Cells, and Cell Cycle Analysis

Cells were seeded on 13 mm cover glass prior to immunofluorescence (IF). Cells were washed in PBS, then fixed in 4% paraformaldehyde for 10 min at RT. They were washed in PBS, then permeabilized and blocked in 3% BSA and 1% serum of the secondary antibody species in PBST (0.1% Triton‐X in PBS). Cells were then incubated ON at 4°C in primary antibodies: DCT (goat, Santa Cruz sc‐10,451, 1:200); c‐Kit (goat, R&D systems AF1356, 1:400); BIII tubulin (Tuj1, mouse, Promega G7121, 1:1000). After 3 × 5 min washes in PBST, cells were incubated in secondary antibodies (Table 12) for 1 h at RT in the dark. Finally, cells were washed 3 × 5 min in PBST, stained with 1 μg/mL DAPI, and mounted on glass slides in ProLong gold antifade. Images were taken using a Nikon A1R confocal microscope.

For cell proliferation analysis by EdU labelling, cells were pulsed with 10 μM EdU for 2 h (melb‐a cells) or 48 h (primary melanocytes) then fixed for IF. Click‐iT EdU reaction mix was added to the cells following secondary antibody incubation and prior to DAPI staining as described by the manufacturer's protocol (ThermoFisher Scientific C10337 or C10339). EdU positive nuclei were quantified using ImageJ.

For cell cycle analysis, successfully selected melb‐a after transduction were incubated with 2 μg/μl (Click‐iT Plus EdU Pacific Blue Flow Cytometry Assay Kit) for 2 h, then fixed and permeabilized following the manufacturer's protocol. Propidium iodide (PI) was used to measure total DNA content. Cells were analyzed using flow cytometry on a BD LSRFortessa Cell Analyzer.

### 4.15 Western Blot

Whole cell lysates were prepared by boiling and shearing cells in 1× Laemmli sample buffer, and protein concentrations were measured using the Qubit protein assay kit (ThermoFisher Scientific Q33212). 5–15 μg of proteins were separated on 4%–15% SDS‐PAGE gels (BioRAD 456‐1084) and transferred onto PVDF membranes. The Spectra multicolor broad range protein ladder (ThermoFisher Scientific 26,634) used as a protein size marker. Western blotting was performed as previously described (Rai et al. 2014). Primary and secondary antibodies are listed in Tables S14 and S15, respectively.

### 4.16 Statistics

Analysis was performed using GraphPad Prism (Version 9.0.1) and presented as mean ± SEM. Differences between two groups were assessed for statistical significance by an unpaired *t‐*test. *p* values ≤ 0.05 were considered significant.

## Author Contributions

F.J.‐H. planned and performed the vast majority of the experiments. R.A. performed melb‐a RNA‐seq, ATAC seq, and differentiation assays and analyses. K.S. provided experimental, computational, and intellectual assistance and advice and optimized melanoblast sorting and scRNAseq with F.J.‐H. K.S., K.G., A.T.W., N.R., and C.H.S. performed single‐cell RNA‐seq analysis. A.L. performed TAF assays. K.K. assisted with single‐cell RNA‐seq. C.R., J.P., T.S.R., and N.F. provided experimental support. J.P. performed experiments with Tyr‐*N‐RAS* ^*Q61K*^ mice. K.B. advised on mouse experiments. J.P.M. and K.Y.Y. advised on single‐cell RNA‐seq analysis. J.F.P. advised on TAF assays. I.B. measured hair pigmentation. L.M.M. and M.L.H. assisted in the direction of the study. P.D.A. and F.J.‐H. conceived and directed the study. F.J.‐H., R.A., and P.D.A. wrote the manuscript. All authors edited the manuscript.

## Conflicts of Interest

The authors declare no conflicts of interest.

## Supporting information


**Figure S1.** (a) Gating strategy for tdTomato+ and c‐KIT+ cell quantification. Gating was performed with FlowJo software and used to quantify Brilliant Violet (BV)711‐CD117 (c‐KIT) positive melanoblasts from tdTomato positive populations of dissociated E15.5 trunk skin cell suspensions. FMO: fluorescence minus one. (b) Representative DCT and BrdU immunofluorescence images in E13.5 and E15.5 embryo cross sections from Hira WT and Hira KO embryos used in quantification of BrdU positive DCT positive cells (Figure 1f). Images were taken using Nikon A1R confocal microscope. Scale bar: 25 μm.
**Figure S2.** Hira is required for melanoblast development. (a) tdTomato fluorescence in FACS isolated cells (samples described in Figure 2a). (b–d) t‐SNE plots from combined three WT samples showing the expression of various melanoblast (c), glial (d), and mesenchymal (e) genes in different clusters as viewed from Loupe Browser 6.0 by10× Genomics. Genes were taken from the lists in Tables S2–S4. (e) 100% stacked column graph displaying the percentage of each cell type within the tdTomato population within each of the six samples described in Figure 2g. (f) Scatter dot plots showing the mean percentages of the major cell populations for each of three HIRA WT and three HIRA KO replicates. Data were analyzed using an unpaired *t‐*test showing mean ± SEM. (g) GSEA showing KO cells enriched for the EMT signature relative to WT cells (NES = 1.43, FDR q = 0.0081).
**Figure S3.** Knockout of HIRA leads to phenotypic and functional disruptions in melb‐a cells. (a) Scatter dot plots representing the average nuclear area of cells in Figure 3a as measured with ImageJ software. (b) Western blot for the HIRA chaperone complex members HIRA, Cabin1, and Asf1a using cell lysates harvested 10 days following infections. (c–g) Western blots for various proteins (lineage markers (c–e), proliferation (e), and histone modifications (f, g)) using cell lysates harvested 10 days following lentiviral infections of melb‐a cells with shLuc, shHira1, and shHira2. Multiple lanes for each represent independent replicates. GAPDH, actin, and LaminA/C were used as sample integrity controls. In panels f and g, the total and modified H3 blots are strip and reprobe of the same membrane/polypeptide. h, Scatter dot plots displaying the average percentage of EdU positive nuclei in melb‐a cells 10 days after infection with shHira1 and shHira2 lentiviral vectors, with shLuc as control. Three independent replicates each (three culture plates each infected separately). Scatter dot plot data in a and f were analyzed using an unpaired *t‐*test showing mean ± SEM. ns: non‐significant (*p* > 0.05). (i) EdU/PI analysis of *Hira* knockdown versus control melb‐a cells. (j) Principal component analysis (PCA) plot of bulk RNA‐seq data. The individual sample replicates have been color‐coded based on the experimental conditions. (k) The top 20 enriched GO:BP terms for the two clusters determined in (d). (l) Top upstream transcription factor analysis of differentially expressed genes in control vs. HIRA KD cells using ingenuity pathway analysis (IPA). (m) Volcano plot of differentially accessible peaks between HIRA KD and control group. (n) The top enriched GO:BP terms for closed TSS peaks in HIRA KD samples. (o) Melanin quantification of melb‐a cells after 9 days of differentiation as measured spectrophotometrically at 405 nm. ns: non‐significant.
**Figure S4.** Knockout of *Hira* does not affect the distribution and number of melanocytes in young adult mice. a, Quantification of signal intensity of H3.3 stain in Figure 4c. (b) Mild difference in hair pigmentation in HIRA KO mice as compared to HIRA WT litter mates at P33 during the first real anagen. (c) DCT/DAPI immunofluorescence and its corresponding phase contrast images showing the presence of pigmented melanocytes in growing hair bulbs of HIRA WT and HIRA KO mouse skin at P33. Scale bar: 20 μm. (d) Representative images of ROI outline on HIRA WT and HIRA KO hair bulbs. **e**, Quantifications of melanin in the hair matrix of anagen hair bulbs in 8–10‐week‐old TyrCre::*HIRA* ^wt/wt^ and TyrCre::*HIRA* ^fl/fl^ female mice. The mean gray value was calculated using ImageJ and scored based on saturation (y‐axis), (black) near 0, and (white) near 255. Analyzed using an unpaired parametric *t*‐test. (f) Maximum and minimum gray values assessed using a Mann–Whitney *U* test. ** indicates a *p*‐value < 0.005. Ten hair bulbs per animal were analyzed across *n* = 3 animals per genotype.
**Figure S5.** No effect after inducible knockout of *Hira* in differentiated melanocytes ex vivo, but suppression of hyperpigmented *TyrNras* phenotype by constitutive *Hira* knockout. (a) UV image of a gel electrophoresis of the EtBr‐stained products of a PCR testing recombination in induced *TyrCreERT2::Hira* ^*fl/fl*^ cells. 4‐OHT‐treated *TyrCreERT2::Hira* ^*wt/wt*^ cells and *Hira* ^*fl/fl*^ cells (without *TyrCreERT2*) were used as floxed allele and recombination controls. Band sizes: 717 bp for WT allele, 910 bp for floxed allele, and 467 bp for recombined allele. First lane: 10‐kb ladder (O’GeneRuler Fermentas SM1173). Second lane: no template control (NTC). (b) Images of a HIRA WT C57Bl6 mouse displaying coat and skin color. (c) Distribution of melanocytes (DCT positive cells in green) in the skins of NRAS‐HIRA WT and NRAS‐HIRA KO p11 pups (representative of *n* = 3 for each genotype). The middle row NRAS‐HIRA KO skin section shows the epidermal and upper dermal regions of the skin, the same region in NRAS‐HIRA WT (top). The bottom row shows the hair bulb region in NRAS‐HIRA KO. Scale bar: 30 μm.
**Figure S6.** Inducible postnatal knockout of *Hira* does not lead to hair graying. (a) Progressive hair graying in aging HIRA KO mice, not observed in HIRA WT mice. (b) Co‐localization of tdTomato (red) and DCT (green) showing melanocyte‐specific recombination in *TyrCreERT2* system in the bulb (Bb) and McSCs (magenta arrow heads) of the bulge (Bg). Images representative of *n* = 4 mice. Scale bar: 25 μm. (c) More images showing recombination in *TyrCreERT2* system in bulge McSCs of telogen (top panel) and anagen (lower panel) hair follicles. Scale bars: 25 μM top; 50 μm bottom. (d) 13‐month‐old HIRA WT and HIRA KO mice after 1 cycle of depilation. (e) Lack of pigment in the Dct + Sox10+ cells in the bulge area of HIRA KO as well as HIRA WT hair follicles, indicating the absence of premature differentiation in McSCs. Representative images of the same were also used in Figure 6h. (f) Only Sox10+ cells in the bulb region of hair follicles are MITF positive, indicating their differentiated melanocyte status, both in HIRA WT and in HIRA KO mice. (g) Sox10+ cells in the bulge are MITF negative in HIRA KO as well as HIRA WT mice, indicating the absence of premature differentiation in HIRA KO McSCs as observed in hair bulges in which McSCs are still present.

**Table S1.** Detailed information on the samples used for single cell RNA sequencing and the cells used for sequencing analysis.
**Table S2.** Top melanoblast distinguishing genes within tdTomato population from 3 WT embryos generated with MAST against all tdTomato population.
**Table S3.** Top glial distinguishing genes within tdTomato population from 3 WT embryos generated with MAST against all tdTomato population.
**Table S4.** Top mesenchymal distinguishing genes within tdTomato population from 3 WT embryos generated using 10× Genomics Loupe browser.
**Table S5.** Number and percentage of cells of the main clusters within the tdTomato population of 3 WT and 3 HIRA KO samples.
**Table S6.** Differentially expressed (DE) genes in HIRA KO vs HIRA WT melanoblasts calculated using MAST R package. Top 50 upregulated and top 50 downregulated genes are shown.
**Table S7.** Differentially expressed (DE) genes in HIRA KO vs HIRA WT glial cells calculated using MAST R package. Top 50 upregulated and top 50 downregulated genes are shown.
**Table S8.** Common HIRA KO vs. HIRA WT differentially expressed (DE) genes between melanoblast and glial cells from top 50 genes.
**Table S9.** Differentially expressed (DE) genes in HIRA KO vs HIRA WT *Sox10*+ cells calculated using MAST R package. Top 50 upregulated and top 50 downregulated genes are shown.
**Table S10.** Differentially expressed (DE) genes in HIRA KD vs HIRA WT melb‐a cells calculated using edgeR. Top 50 upregulated and top 50 downregulated genes are shown.
**Table S11.** Primary antibodies used in IHC.
**Table S12.** Secondary antibodies used in IHC.
**Table S13.** Technical information on samples used in scRNA seq.
**Table S14.** Primary antibodies used in western blotting.
**Table S15.** Secondary antibodies used in Western blotting.

## Data Availability

scRNAseq data were deposited in the Gene Expression Omnibus (GEO) and can be accessed using reviewer token gjmdcmimzdollol through the following link: https://www.ncbi.nlm.nih.gov/geo/query/acc.cgi?acc=GSE132545. The GEO record for RNA‐seq can be accessed here: https://www.ncbi.nlm.nih.gov/geo/query/acc.cgi?acc=GSE267432 and that for ATAC seq can be accessed here: https://www.ncbi.nlm.nih.gov/geo/query/acc.cgi?acc=GSE267431 using reviewer token uxypmmeaxdmdjgr. All raw data used to generate the results and figures of this study are available upon request. This includes scripts used for scRNAseq analysis, original IHC and IF images, quantification of dermal pigmentation, original Western blot scans, and individual cell counts for melanoblasts, melanocytes, and McSCs (DCT‐LacZ, BrdU, EdU, DCT immunofluorescence, TAFs, and flow cytometry).
