# Supplementary figures and images for: Inactivation of Histone Chaperone HIRA Unmasks a Link Between Normal Embryonic Development of Melanoblasts and Maintenance of Adult Melanocyte Stem Cells
Source: Aging Cell. 2025 May 14;24(7):e70070. doi: 10.1111/acel.70070 (PMC12266762; doi:10.1111/acel.70070)

a

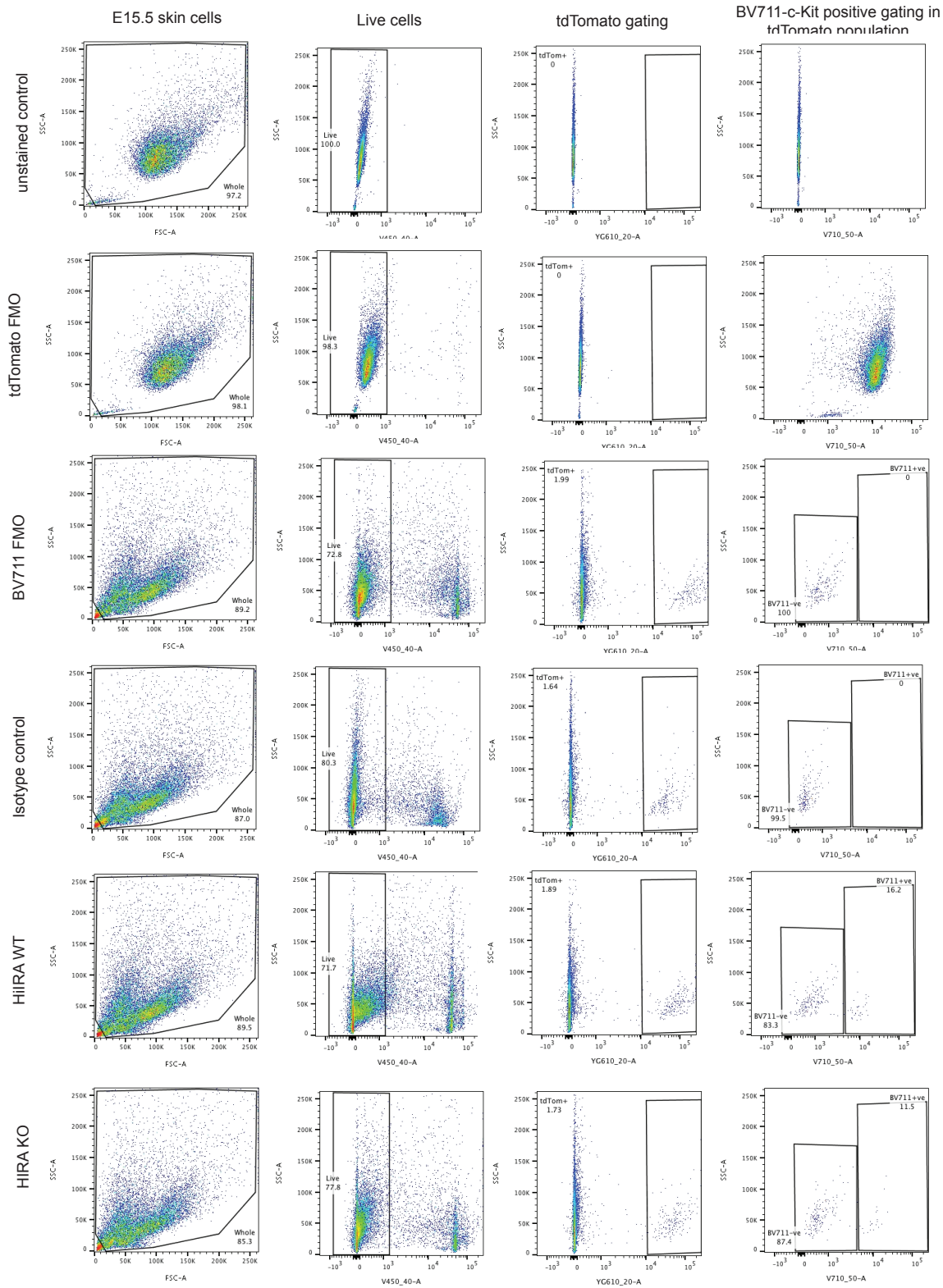

b

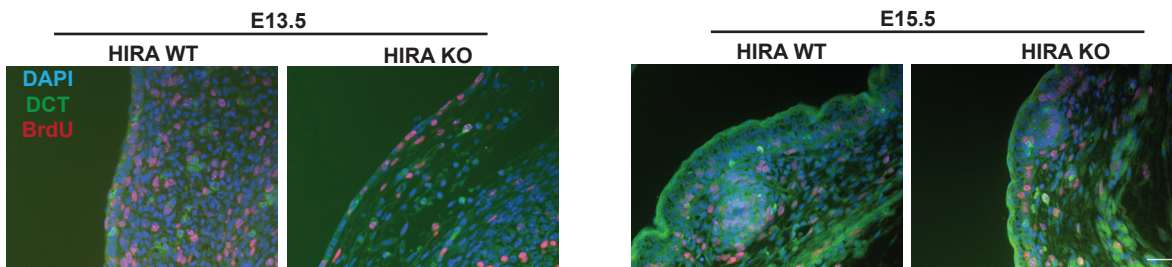

a

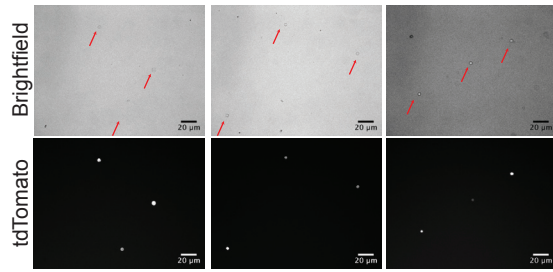

b

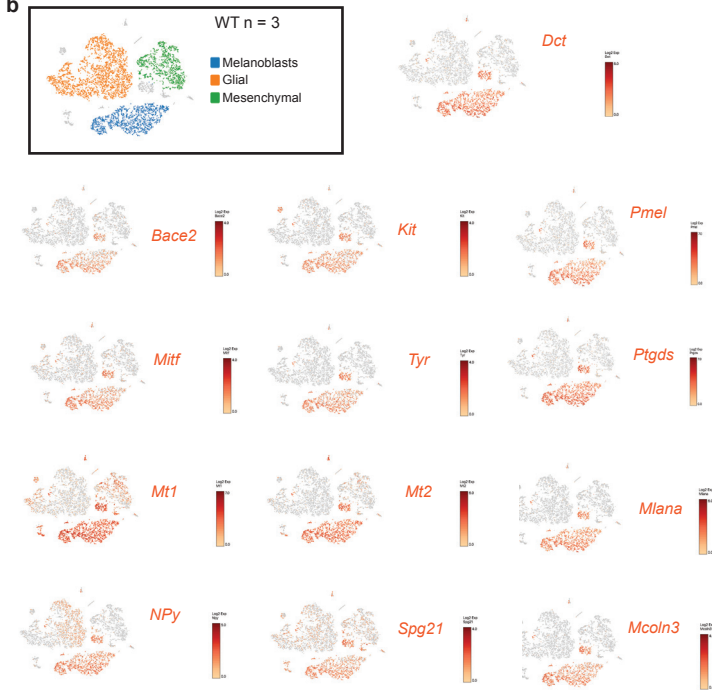

c

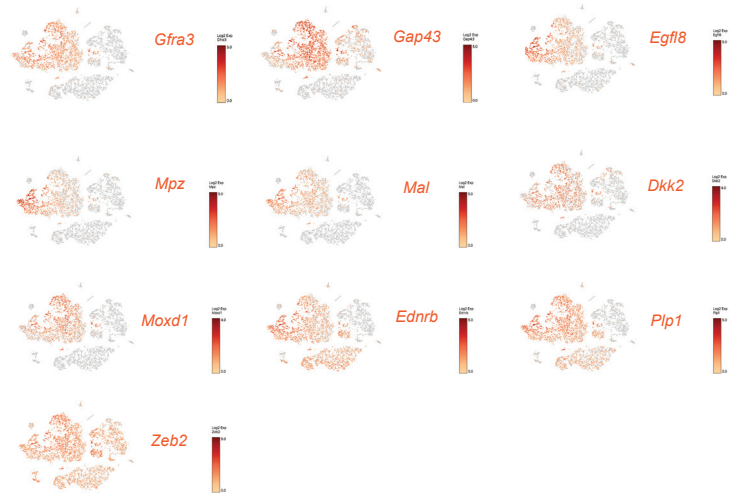

d

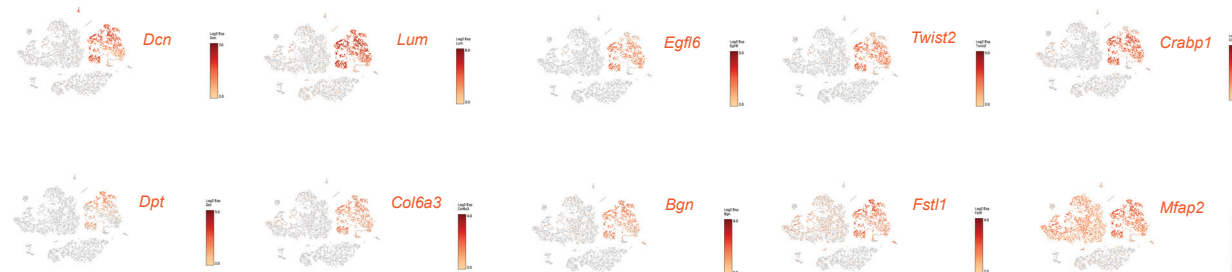

e

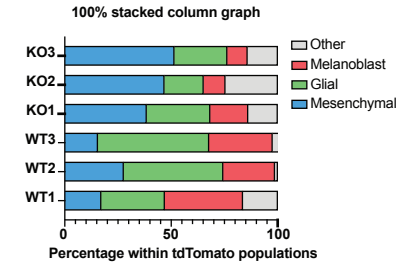

f

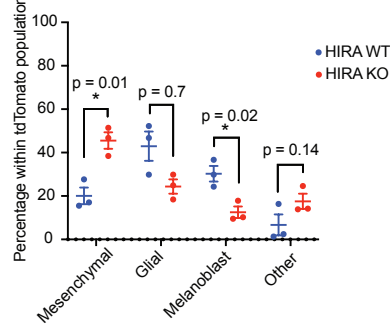

g

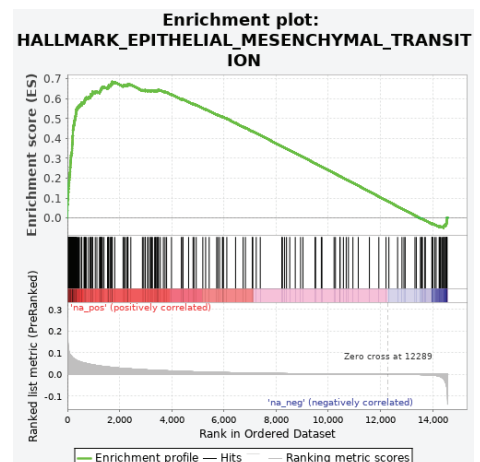

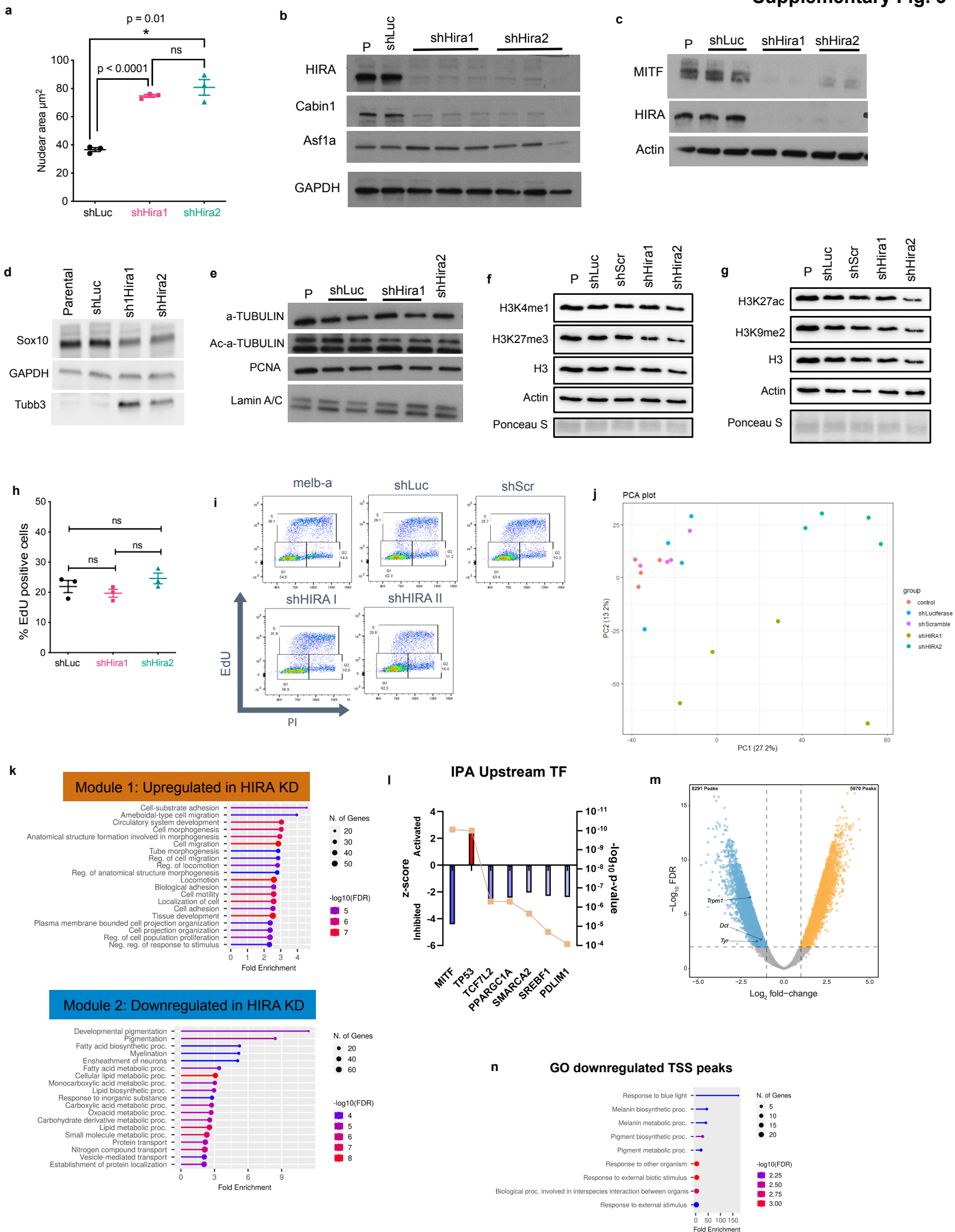

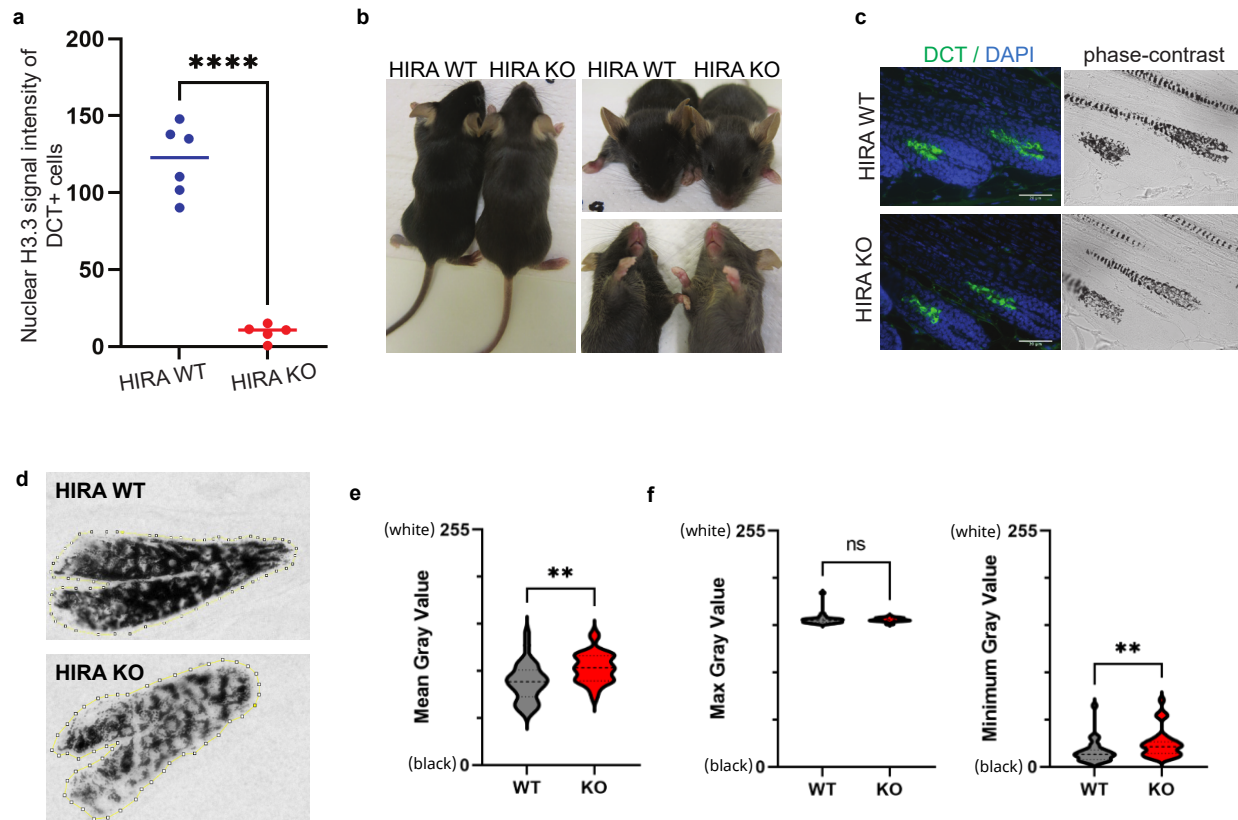

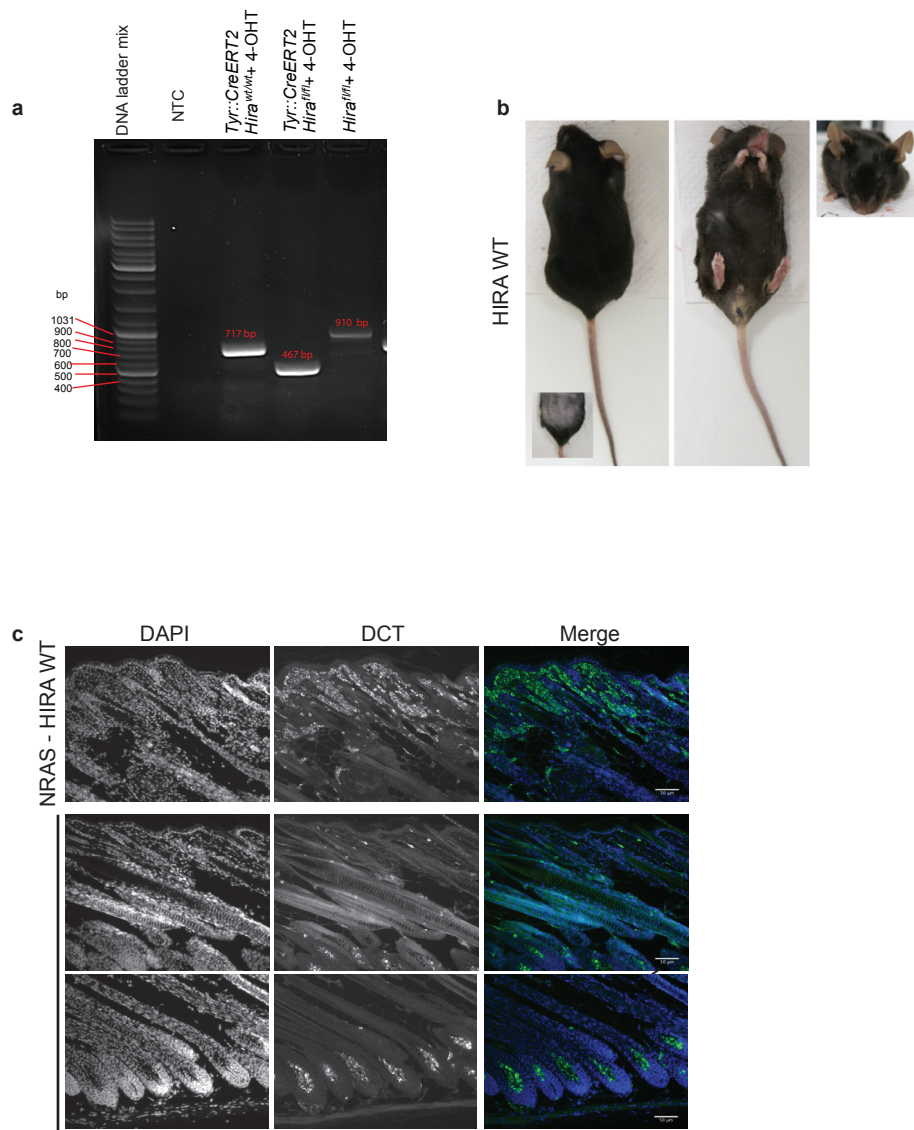

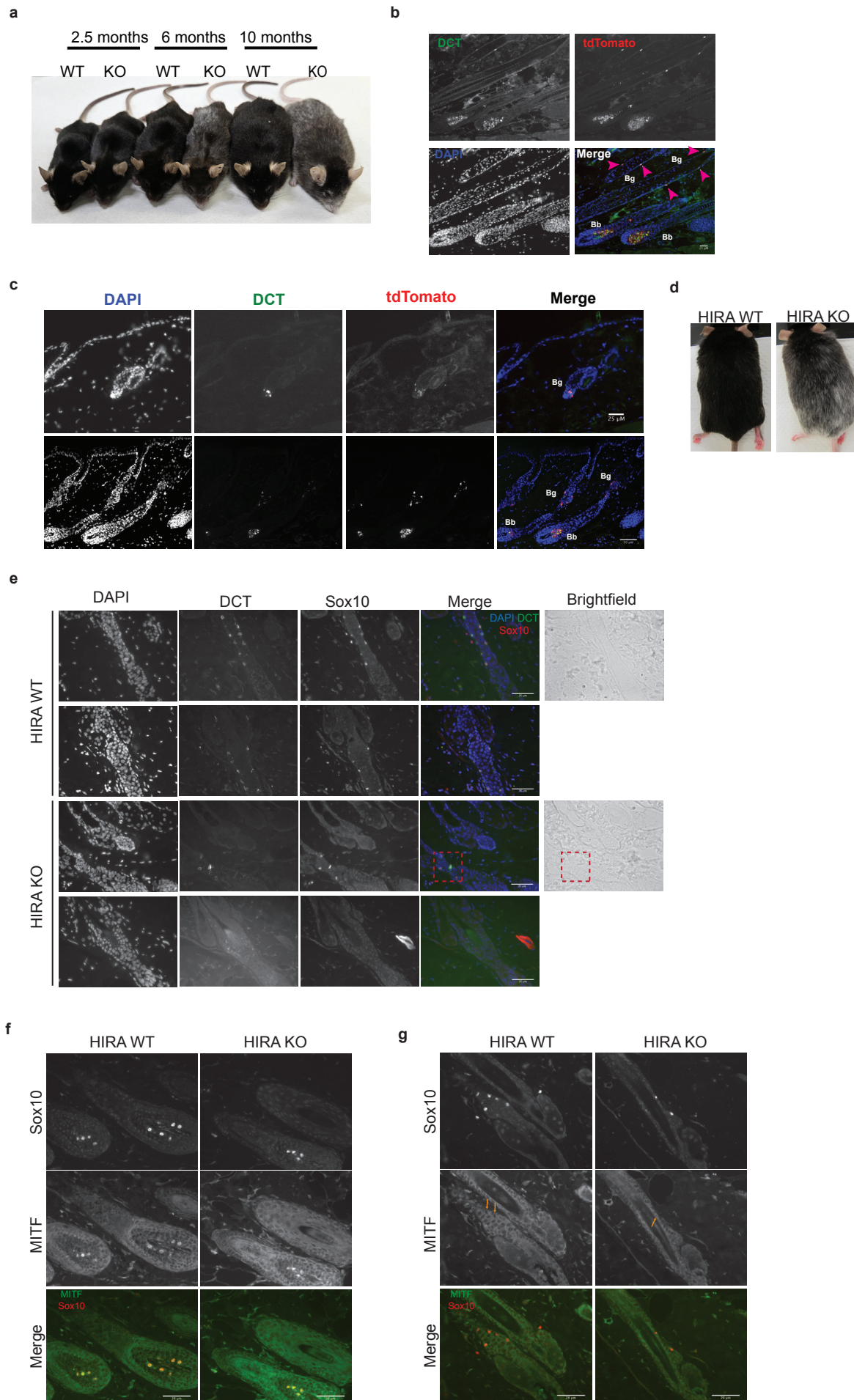

Supplement: Supplementary file 1 — Figure S1. (a) Gating strategy for tdTomato+ and c‐KIT+ cell quantification. Gating was performed with FlowJo software and used to quantify Brilliant Violet (BV)711‐CD117 (c‐KIT) positive melanoblasts from tdTomato positive populations of dissociated E15.5 trunk skin cell suspensions. FMO: fluorescence minus one. (b) Representative DCT and BrdU immunofluorescence images in E13.5 and E15.5 embryo cross sections from Hira WT and Hira KO embryos used in quantification of BrdU positive DCT positive cells (Figure 1f). Images were taken using Nikon A1R confocal microscope. Scale bar: 25 μm. Figure S2. Hira is required for melanoblast development. (a) tdTomato fluorescence in FACS isolated cells (samples described in Figure 2a). (b–d) t‐SNE plots from combined three WT samples showing the expression of various melanoblast (c), glial (d), and mesenchymal (e) genes in different clusters as viewed from Loupe Browser 6.0 by10× Genomics. Genes were taken from the lists in Tables S2–S4. (e) 100% stacked column graph displaying the percentage of each cell type within the tdTomato population within each of the six samples described in Figure 2g. (f) Scatter dot plots showing the mean percentages of the major cell populations for each of three HIRA WT and three HIRA KO replicates. Data were analyzed using an unpaired t‐test showing mean ± SEM. (g) GSEA showing KO cells enriched for the EMT signature relative to WT cells (NES = 1.43, FDR q = 0.0081). Figure S3. Knockout of HIRA leads to phenotypic and functional disruptions in melb‐a cells. (a) Scatter dot plots representing the average nuclear area of cells in Figure 3a as measured with ImageJ software. (b) Western blot for the HIRA chaperone complex members HIRA, Cabin1, and Asf1a using cell lysates harvested 10 days following infections. (c–g) Western blots for various proteins (lineage markers (c–e), proliferation (e), and histone modifications (f, g)) using cell lysates harvested 10 days following lentiviral infections of melb‐ [file ACEL-24-e70070-s001.pdf]
